# CDK16 promotes the progression and metastasis of triple-negative breast cancer by phosphorylating PRC1

**DOI:** 10.1186/s13046-022-02362-w

**Published:** 2022-04-21

**Authors:** Xiao Li, Jinpeng Li, Liming Xu, Wei Wei, Anyi Cheng, Lingxian Zhang, Mengna Zhang, Gaosong Wu, Cheguo Cai

**Affiliations:** 1 Department of Thyroid and Breast Surgery, Frontier Science Center for Immunology and Metabolism, Medical Research Institute, Zhongnan Hospital of Wuhan University, Wuhan University, Wuhan, 430071 China; 2 Department of Thyroid and Breast Surgery, Zhongnan Hospital of Wuhan University, Wuhan, 430071 China

**Keywords:** Atypical CDK, CDK16, Breast cancer, TNBC, PRC1

## Abstract

**Background:**

Cyclin-dependent kinase 16 (CDK16) is an atypical PCTAIRE kinase, and its activity is dependent on the Cyclin Y (CCNY) family. Ccnys have been reported to regulate mammary stem cell activity and mammary gland development, and CCNY has been recognized as an oncoprotein in various cancers, including breast cancer. However, it remains unclear whether CDK16 has a role in breast cancer and whether it can be used as a therapeutic target for breast cancer.

**Methods:**

Publicly available breast cancer datasets analyses and Kaplan-Meier survival analyses were performed to reveal the expression and clinical relevance of atypical CDKs in breast cancer. CDK16 protein expression was further examined by immunohistochemical and immunoblot analyses of clinical samples. Cell proliferation was measured by colony formation and MTT analyses. Cell cycle and apoptosis were examined by fluorescence-activated cell sorting (FACS) analysis. Wound-healing and trans-well invasion assays were conducted to test cell migration ability. The functions of CDK16 on tumorigenesis and metastasis were evaluated by cell line-derived xenograft, patient-derived organoid/xenograft, lung metastasis and systemic metastasis mouse models. Transcriptomic analysis was performed to reveal the potential molecular mechanisms involved in the function of CDK16. Pharmacological inhibition of CDK16 was achieved by the small molecular inhibitor rebastinib to further assess the anti-tumor utility of targeting CDK16.

**Results:**

CDK16 is highly expressed in breast cancer, particularly in triple-negative breast cancer (TNBC). The elevated CDK16 expression is correlated with poor outcomes in breast cancer patients. CDK16 can improve the proliferation and migration ability of TNBC cells in vitro, and promote tumor growth and metastasis of TNBC in vivo. Both genetic knockdown and pharmacological inhibition of CDK16 significantly suppress the tumor progression of TNBC. Mechanistically, CDK16 exerts its function by phosphorylating protein regulator of cytokinesis 1 (PRC1) to regulate spindle formation during mitosis.

**Conclusion:**

CDK16 plays a critical role in TNBC and is a novel promising therapeutic target for TNBC.

**Supplementary Information:**

The online version contains supplementary material available at 10.1186/s13046-022-02362-w.

## Background

Breast cancer is the most common malignancy and the leading cause of cancer-associated death in women [1]. According to immunohistochemistry (IHC) assessment of estrogen receptor (ER), progesterone receptor (PR), and human epidermal growth factor-2 (HER2), together with clinicopathological features, breast cancer can be divided into the following subtypes: luminal A (ER^+^ and/or PR^+^, HER2^−^, low Ki67), luminal B (ER^+^ and/or PR^+^, high Ki67 or HER2^+^), HER2-amplified (HER2^+^, ER^−^ and PR^−^), and triple-negative breast cancer (TNBC) (ER^−^, PR^−^, and HER2^−^) [2]. TNBC accounts for 15–20% of diagnosed breast cancer, and is the most refractory subtype with the highest risk of recurrence and mortality [3]. ER^+^ and HER2^+^ breast cancer patients have achieved great therapeutic benefits through endocrine therapy (e.g., aromatase inhibitors, SERMs, and SERDs) [4] and anti-HER2 therapeutic antibodies (e.g., trastuzumab and pertuzumab) [5], respectively. However, due to high genetic heterogeneity [6] and lack of targetable molecules, targeted therapy of TNBC is still challenging, and chemotherapy remains the routine treatment [7]. Therefore, it is urgent to reveal the critical carcinogenic factors of TNBC, discover novel therapeutic targets, and develop effective targeted drugs for TNBC, to improve the clinical outcomes of TNBC patients.

Cyclin-dependent kinases (CDKs) are a large family of proline-directed serine/threonine protein kinases characterized by the need for a regulatory subunit, a cyclin, to perform the enzymatic activity [8]. The mammalian CDK family consists of 21 members (CDK1–20, CDK11 has two isoforms CDK11A/B) [9]. CDKs regulate cell cycle progression (CDK1, 2, 3, 4, 6) and transcription events (CDK7–13, 19, 20) in response to extracellular and intracellular signals leading to cell proliferation [10, 11]. Since uncontrolled cell proliferation is a hallmark of cancer [12], targeting dysregulated CDK in specific cancers to prevent tumor cell proliferation has become an effective anti-cancer therapeutic strategy. For example, CDK4/6 inhibitors have been developed and approved for the treatment of advanced or metastatic ER^+^/HER2^−^ breast cancer due to the essential role of CDK4/6 in cell cycle regulation [13]. Moreover, CDK7 [14] and CDK12/13 [15] have been identified as potential therapeutic targets for TNBC because of their transcriptional regulation of genes crucial to the survival of tumor cells.

Aside from the typical CDKs involved in cell cycle and transcriptional regulation, there is a distinct subfamily of CDKs with a close evolutionary relationship and unclear function description. These CDKs are sorted into atypical CDKs, including CDK5, the newly proposed PFTAIRE (PFTK1, 2, also known as CDK14, 15) and PCTAIRE (PCTK1–3, also known as CDK16–18) kinases [11]. PFTAIRE and PCTAIRE kinases have been less studied as they were once considered “orphan CDKs” until their cyclin partners-Cyclin Y (CCNY) and Cyclin Y-like 1 (CCNYL1) were identified [16]. The oncogenic role of CCNY has been reported in various cancers, including breast cancer [17–20], suggesting the potential function of CCNY-dependent CDKs in cancer progression.

A notable study showed that inhibition of the 5hmC epigenetic modification in CCNY/CDK16 promoters impaired the activity of renal cancer stem cells, suggesting the potential effects of CDK16 on cancer stem cells (CSCs) [21]. Our previous studies have found that Ccny and Ccnyl1 play an essential role in regulating mammary stem cell activity and mammary development [22]. These findings prompted us to further explore whether CCNY-dependent CDKs could be ideal targets for breast cancer therapy. In this study, we sought to reveal a novel therapeutic target for breast cancer among CCNY-dependent CDKs by clinical correlation analysis and functional study. We found that CDK16 exhibits a distinct expression pattern in breast cancer and has strong clinical relevance, making it a potential therapeutic target for breast cancer, especially TNBC. In support of this notion, we described the oncogenic role of CDK16 in TNBC, assessed the therapeutic potential of CDK16 as a target in multiple TNBC models, and elucidated the molecular mechanisms of CDK16 involved in TNBC progression. Our study provides new insights into the roles of atypical CDKs in cancer, highlights the significance of CDK16 in breast cancer, especially TNBC, and offers a promising target for TNBC therapy.

## Methods

### Patient samples

Clinical samples used in this study were obtained from Zhongnan Hospital of Wuhan University.

### Experimental animals

BALB/c, Nude, and SCID/Beige mouse strains used in our study were purchased from Charles River Laboratories or Gem Pharmatech and maintained under specific-pathogen-free (SPF) conditions.

### Cell culture and lentivirus transfection

Cell lines used in this study were obtained from American Type Culture Collection (ATCC). Cells were maintained in appropriate medium supplemented with 10% fetal bovine serum (FBS) (PAN-Biotech) and 1% penicillin/streptomycin (PS) (HyClone) at 37 °C incubators with 5% CO_2_.

For lentivirus preparation, constructed plasmids were co-transfected with psPAX2 (Addgene_12260) and pMD2.G (Addgene_12259) at a 4:3:2 mass ratio into HEK293T cells using polyetherimide (PEI). Two days after transfection, the virus particles were collected and filtered through 0.45 μm filters. Cells were infected and sorted by FACS (FACSAria III, Becton, Dickinson and Company) to generate stable lines.

### Plasmid construction

Human *CDK16* (NM_006201.5), mouse *Cdk16* (NM_011049.5) and human *PRC1* (NM_006201.5) were cloned by PCR and constructed in the pWPI-EGFP (Addgene_12254) vectors for overexpression. *CDK16*^*D304A*^, *PRC1*^*T481D*^, and *PRC1*^*T481A*^ mutants were generated by a PCR-based site-directed mutagenesis method. shRNA sequences were synthesized by Sangon Biotech Co., Ltd. (Shanghai) then cloned into pLKO.1-GFP backbone, and the sequences are listed as follows:

Scramble control: 5′-CAACAAGATGAAGAGCACCAA-3′.

*CDK16*-sh1: 5′-GACCTACATTAAGCTGGACAA-3′.

*CDK16*-sh2: 5′-CGAGGAGTTCAAGACATACAA-3′.

*CDK16*-sh3: 5′-GCTCTCATCACTCCTTCACTT-3′.

*Cdk16*-sh: 5′-GCACTAAAGGAGGTACAGCTA-3′.

### RNA extraction, reverse transcription and quantitative real-time PCR (RT-qPCR)

Total RNA was extracted with RNAiso Plus (TaKaRa) and cDNA was obtained by reverse transcription using PrimeScript RT Master Mix (TaKaRa). Quantitative real-time PCR (qPCR) was performed with FastStart Universal SYBR Green Master Mix (Monad) on a CFX Connect Real-Time PCR Detection System (Bio-Rad). Primers used for qPCR analyses are listed in Supplementary Table S1, and GAPDH was used as a reference in all analyses.

### Western blotting and cellular fractionation

Total cell lysates were obtained using RIPA buffer (CWBIO) containing protease inhibitor PMSF (Selleck) and dithiothreitol (DTT) (Sigma). Proteins were separated by SDS-PAGE and transferred to PVDF membranes (Millipore). The bands were visualized using the ECL chemiluminescence kit (Monad). Nuclear-cytoplasmic fractions were obtained using the Nuclear and Cytoplasmic Protein Extraction Kit (catalog no. P0027; Beyotime), then subjected to western blotting. Antibodies used are shown in Supplementary Table S2.

### Immunofluorescence (IF) analysis

Cells were seeded on coverslips in 24-well plates and grew for 24 h. Then, cells were fixed in 4% paraformaldehyde (PFA) for 30 min, permeabilized with 0.2% Triton X-100 for 30 min, and blocked with 10% goat serum in PBS for 2 h, followed by incubation with specific primary antibodies (overnight, 4 °C) and with fluorochrome-conjugated secondary antibodies (2 h, RT). Before imaging, coverslips were mounted using ProLong Gold Antifade Reagent with DAPI (catalog no. P36931; Thermo Fisher Scientific). Immunostaining of tumor organoid smears and tumor tissue sections was also performed as described above. Images were acquired using a laser scanning microscope (LSM880, ZEISS) and analyzed with the ZEN (blue edition) 2.3 software (ZEISS). Antibodies used are listed in Supplementary Table S2.

### Immunohistochemistry (IHC) analysis

Breast cancer tissue microarray (F551101) was purchased from Zhongke Guanghua (Xi’an) Intelligent Biotechnology Co., Ltd. The slide was first deparaffinized and rehydrated. After antigen retrieval with boiling sodium citrate buffer (10 mM, pH 6.0), the slide was incubated with 3% H_2_O_2_, and blocked with 10% goat serum albumin, followed by incubation with CDK16 antibody (catalog no. HPA001366; Sigma-Aldrich) (4 °C, overnight) and goat anti-rabbit HRP secondary antibody (30 min, RT). Subsequently, the slide was stained with 3,3′-diaminobenzidine (DAB) and counterstained with hematoxylin. CDK16 expression was semi-quantitatively assessed by H-score (HS, range 0 ~ 300), which was calculated as the intensity score (0, negative; 1, faint/weak; 2, moderate; 3, strong) multiplied by the distribution score (between 1 [percentage] to 100 [percentage]). H-score reading was conducted by three examiners who were blinded to clinical data and the results of each other. Subsequently, stratification scoring (IHC score) was performed according to HS: HS < 80, scored as 0; 80 < HS < 120, scored as 1; 120 < HS < 200, scored as 2; HS > 200, scored as 3.

### Hematoxylin-eosin (HE) staining

Paraffin slides were deparaffinized and rehydrated, then stained with hematoxylin solution for 5 min, differentiated in 1% acid alcohol solution, followed by eosin staining for 2 min. After dehydration and hyalinization, the slides were mounted and imaged.

### Cell viability assay

Cell viability was measured by 3-(4,5-dimethylthiazol-2-yl)-3,5-diphenyltetrazolium bromide (MTT) assay. Cells were seeded in 96-well plates (10,000 ~ 20,000 cells/well). After growth for 12 h, increasing concentrations of rebastinib was supplemented and incubated for 72 h. MTT was added and incubated for additional 4 h, followed by the addition of DMSO to dissolve the Formazan. Cell viability was measured by OD570nm using a microplate reader (SpectraMax i3x, Molecular Devices).

### Colony formation assay

For 2D-plate colony formation assay, cells were seeded in triplicate in 6-well plates (500 ~ 1000 cells/well) and cultured for about 2 weeks. Colonies were counted following crystal violet staining. For 3D-soft agar colony formation assay, 6-well plates were precoated with growth medium containing 1% low melting agarose (catalog no. A600015; Sangon Biotech). Cells were suspended in growth medium with 0.4% agarose and placed onto the bottom layer in triplicate (20,000 ~ 50,000 cells/well). Cells were cultured for about 4 weeks with several drops of fresh medium added every 4 days. Colonies were counted following staining with 100 mg/ml iodonitrotetrazolium chloride (catalog no. A610280; Sangon Biotech) solution overnight.

### Migration and invasion assay

For wound-healing assay, cells were seeded in 6-well plates (1 × 10^5^ cells/ well) to monolayer confluent and starved overnight, then scratches were made by a 200 μl pipette tip. Cells were washed with PBS to remove detached cells, and supplemented with appropriate growth medium with 1% FBS. The area within the scratch was measured using ImageJ software at once (0 h) and after 12 h (12 h), and the migrated area was defined as the difference of area within the scratch between 0 h and 12 h. For trans-well invasion assay, transwells with 8 μm pore size polycarbonate membranes were pre-coated with Matrigel (catalog no. 354230; Corning). Two hundred microlitre of serum-free cell suspension (3 × 10^4^ cells per insert) was placed in the upper chamber, and the medium with 20% FBS was supplemented to the lower chamber. After incubation at 37 °C for 24 h, cells were fixed with 4% PFA and stained with 0.1% crystal violet, then cells migrated to the lower side of the filter were imaged and counted.

### Cell cycle and cell apoptosis analyses

Cell cycle and cell apoptosis analyses were conducted using the DNA Content Quantitation Assay Kit (catalog no. CA1510; Solarbio) and AnnexinV-PI apoptosis assay kit (catalog no. FXP023; 4A BIOTECH) following the manufacturer’s instructions. Data were acquired on a flow cytometer (LSRFortessaX-20, Becton, Dickinson and Company) and analyzed by Modfit LT5.0 or FlowJo V10 software.

### Patient-derived xenograft (PDX) model

Fresh tumor fragments from breast cancer patients were subcutaneously transplanted into 4-week female SCID/Beige mice, and the established PDX was maintained by serial passage in nude mice. Each generation was confirmed the histological and biomarker fidelity with their tumors of origin. Tumor cells were dissociated from established xenografts and infected with scramble or *CDK16*-shRNA lentivirus in cell suspension, then inoculated into the mammary fat pads of nude recipient mice for tumorigenesis assay.

### Patient-derived organoid (PDO) model

Tumor cells dissociated from clinical specimens were infected with scramble or *CDK16*-shRNA lentivirus, then suspended in chilled growth-factor-reduced Matrigel (catalog no. 354230; Corning), and pipetted 50 μl drops into each well of 24-well plates (1 × 10^5^ cells/well), subsequently submerged the 3D cultures with modified M87 culture medium (formulated as described in [23]). Cells were cultured for about 7 days with culture medium renewed every 2 days. For inhibitor treatment, indicated concentrations of rebastinib (catalog no. HY-13024; MCE) or abemaciclib (catalog no. HY-16297; MCE) was supplemented at the third day and treated for 48 h. When organoids were ready to harvest, Matrigel was digested by incubation with dispase (catalog no. 354235; Corning) (37 °C, 30 min) to release organoids, then the number and size of the organoids were analyzed.

### Mouse xenograft model

For orthotopic transplantation, cells were suspended in the mixture (1:1, v/v) of 50% FBS/PBS and Matrigel, and implanted into the 4th mammary fat pads (2 × 10^5^ cells/fat pad for MDA-MB-231 and 3 × 10^5^ cells/fat pad for PDX line) of 6-week female nude mice. For subcutaneous transplantation, MDA-MB-468 cells were suspended in PBS and injected subcutaneously into the axillae (2 × 10^6^ cells/flank) of 6-week female nude mice. Tumor growth was monitored, and tumor size was measured every 2 or 3 days. Tumor volume was calculated by the formula: V(volume) = L(length) × W(width)^2^ × 0.52.

### Mouse metastasis model

For lung metastasis model, MDA-MB-231-Luc cells were suspended in PBS and injected into nude recipients via lateral tail vein (5 × 10^5^ cells/mouse for CDK16-OE analysis and 1 × 10^6^ cells/mouse for rebastinib administration). The lung metastasis was monitored by bioluminescence imaging (BLI) at regular intervals. Mice were given the substrate D-luciferin (150 mg/kg) by intraperitoneal injection, then subjected to BLI using IVIS Spectrum instrument. Metastatic burdens were measured using AMIView software. At the experimental endpoint, the lungs were dissected, fixed and dehydrated, then the metastatic nodules on the lung surface were counted. To clearly present minor metastases, the lungs were stained with India ink, excised and decolorized before counting lung nodules. For systemic metastasis model, 4T1-Luc or EMT6-Luc cells were inoculated into the left cardiac ventricles of BALB/c recipients (1 × 10^5^ cells/mouse for both 4T1 and EMT6) and the systemic dissemination of tumor cells was also detected by BLI.

### Administration of rebastinib

To test the effect of rebastinib on tumor growth of TNBC, mice bearing MDA-MB-231 tumors or PDX tumors were randomly grouped when the average tumor size reached 50 mm^3^. Rebastinib (80 mg/kg) formulated in 0.5% hydroxypropyl methyl cellulose (HPMC) or vehicle were orally administrated once a day for over 10 days.

To test the effect of rebastinib on tumor metastasis of TNBC, mice were inoculated intravenously of MDA-MB-231-Luc cells and randomly grouped. Daily oral dosing of rebastinib at 50 mg/kg or vehicle started the next day and lasted for 3 weeks. In vivo lung metastasis was continuously monitored for another week after rebastinib withdrawal.

### TCGA, GEO, CPTAC data analysis and Kaplan-Meier survival analysis

The mRNA expression levels of atypical CDKs in various subtypes of breast cancer were analyzed using clinical data from The Cancer Genome Atlas (TCGA) database (https://portal.gdc.cancer.gov/), METABRIC, Nature 2012 & Nat Commun 2016 combined databases (http://www.cbioportal.org/datasets) and Gene Expression Omnibus (GEO) profiles (https://www.ncbi.nlm.nih.gov/geoprofiles/). Z-score or log2 scale normalized data of individual atypical CDK mRNA expression were extracted, and the expression differences between normal tissues and tumors were analyzed. The correlation between CDK16 expression and clinical outcomes were further analyzed by Kaplan-Meier analysis (the expression data were stratified into high or low by best cutoff using surv_cutpoint function in survminer package). The expression of CDK16 protein in breast cancer samples from the Clinical Proteomic Tumor Analysis Consortium (CPTAC) dataset was analyzed using UALCAN portal (http://ualcan.path.uab.edu/index.html). In addition, the clinical relevance of individual atypical CDKs (CDK14/15/17/18) was explored using public clinical database Kaplan-Meier Plotter of breast cancer (parameter: 120 months of follow-up time and best cutoff). We assessed the correlation between the mRNA expression of CDK14/15/17/18 and the overall survival (OS), recurrence-free survival (RFS), and distant metastasis-free survival (DMFS) of breast cancer patients with the latest version of Kaplan-Meier Plotter database (2021 version, http://www.kmplot.com/analysis/index.php?p=service).

### Transcriptomic analysis

Total RNA samples were submitted to Wuhan Seqhealth Co., Ltd. and RNA sequencing (RNA-seq) was performed on Hiseq X 10 sequencer (Illumina). RNA reads were mapped and RPKMs were calculated. Differentially expressed genes (DEG) were defined with FDR corrected *p*-value≤0.05 and Fold-change≥2 using the edgeR package. Gene Ontology (GO) and Kyoto Encyclopedia of Genes and Genomes (KEGG) analyses of DEG were implemented by the clusterProfiler R package. Gene Set Enrichment Analysis (GSEA) was performed using GSEA software.

### Statistical analysis

Data analyses were performed using GraphPad Prism 8 software. Data were presented as mean ± SD or mean ± SEM unless specified otherwise. *p* values were obtained by unpaired Student’s *t*-test or two-way ANOVA, and *p* ≤ 0.05 was considered statistically significant. The in vitro experiments were repeated independently three times with consistent results.

## Results

### CDK16 is highly expressed in TNBC and is correlated with poor clinical outcomes

**Fig. 1:**
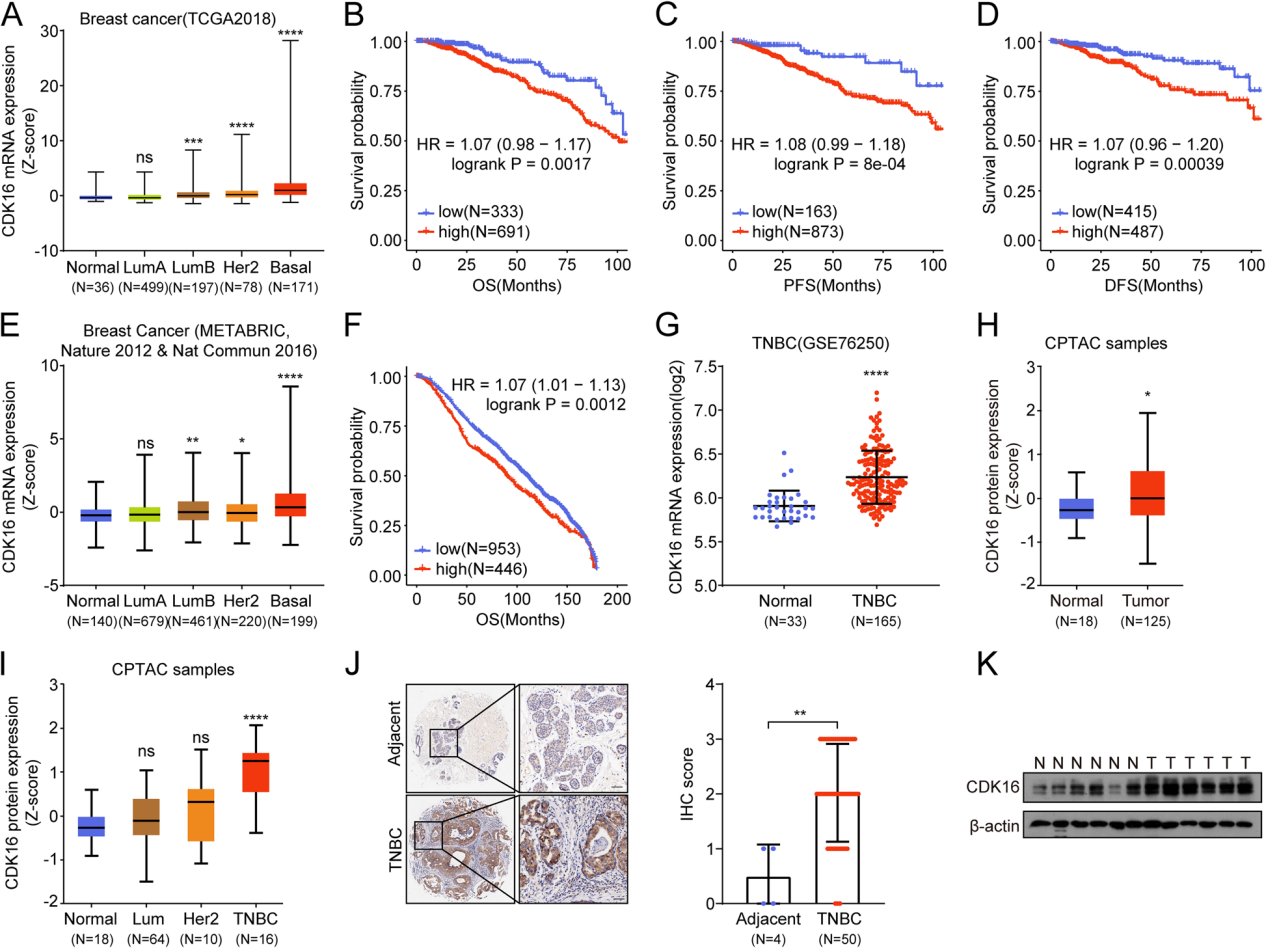
CDK16 is highly expressed in TNBC and is correlated with poor clinical outcomes. **A-D** Clinical relevance analysis of CDK16 using data from TCGA breast cancer dataset. Shown are *CDK16* mRNA expression profile in different molecular subtypes of breast cancer (**A**) and Kaplan-Meier curves of OS (**B**), PFS (**C**), and DFS (**D**) of breast cancer patients stratified by *CDK16* mRNA levels. **E-F** Clinical relevance analysis of CDK16 using data from combined databases of METABRIC, Nature 2012 and Nature Communication 2016. Shown are *CDK16* mRNA expression pattern (**E**) and overall survival analysis (**F**). **G** *CDK16* mRNA expression level in normal breast tissues and in TNBC tissues of patients in GSE76250 dataset. **H-I** CDK16 protein expression in normal and breast cancer tissues (**H**), and in distinct breast cancer subtypes (**I**) of CPTAC samples. **J** Representative immunohistochemical staining images of TNBC and adjacent tissues elucidated by CDK16 antibody (left panel, scale bar: 50 μm) and statistical analysis of immunohistochemical staining intensity (right panel). The commercial tissue microarray used in (J) was from Zhongke Guanghua (Xi’an) Intelligent Biotechnology Co., Ltd. **K** Immunoblot analysis of CDK16 protein expression in clinical non-cancerous (N) and TNBC (T) samples collected from Zhongnan Hospital of Wuhan University. Error bars represent mean ± SD. *p* values were obtained by log rank test (**B, C, D,** and **F**) or two-tailed Student’s *t*-test (others). All *p < 0.05, ** *p* < 0.01, ***p < 0.001, **** *p* < 0.0001, ns, not significant

First, we analyzed the expression (Fig. S1A, C, E, G) and clinical correlations (Fig. S1B, D, F, H) of CCNY-dependent atypical CDKs (*CDK14, CDK15, CDK16, CDK17,* and *CDK18*). Our analysis showed that *CDK16* was expressed in all breast cancer subtypes and the highest expression was found in basal-like breast cancer samples (Fig. 1A). In addition, high expression of *CDK16* was significantly correlated with poor prognosis, as shown by lowered overall survival (OS), progression-free survival (PFS) and disease-free survival (DFS) (Fig. 1B-D). Consistent with the results from TCGA dataset, Breast Cancer (METABRIC, Nature 2012 & Nature Commun 2016) combined databases [24–26] (Fig. 1E) and GSE76250 dataset (Fig. 1G) also revealed that *CDK16* expression was elevated in TNBC compared with normal breast tissues, and the high *CDK16* expression was positively correlated with poor clinical outcomes (Fig. 1F).

We further investigated the CDK16 protein expression in breast cancer. CPTAC database analysis revealed that CDK16 protein expression was significantly elevated in breast tumors compared to normal breast tissues (Fig. 1H). Moreover, among all breast cancer subtypes, CDK16 was exclusively elevated in TNBC (Fig. 1I). To verify the expression of CDK16 protein, we performed immunohistochemical (IHC) analysis using a tissue microarray containing 50 TNBC tumor tissues and four adjacent tissues. Consistent with the results from the CPTAC database, IHC analysis showed that the expression of CDK16 protein in TNBC tumors was significantly higher than that in adjacent non-cancerous tissues, and CDK16 was mainly distributed in the cytoplasm (Fig. 1J). We collected additional six cases of clinical TNBC tumor samples and an equal number of non-cancerous samples, and examined the protein expression of CDK16 by immunoblot analysis. The results further confirmed the increased CDK16 protein expression in TNBC (Fig. 1K). Taken together, these data demonstrated that highly expressed CDK16 may play a role in the progression of TNBC.

### Knockdown of CDK16 inhibits tumor growth of TNBC in vitro and in vivo

We next investigated the functional role of CDK16 in TNBC progression in vitro and in vivo. *CDK16* was knocked down (CDK16-KD) in representative TNBC cell lines (MDA-MB-231, MDA-MB-468 and HCC1937) using two separate shRNA sequences targeting *CDK16*. CDK16 was efficiently knocked down, as confirmed by western blot analysis (Fig. S2A).

**Fig. 2:**
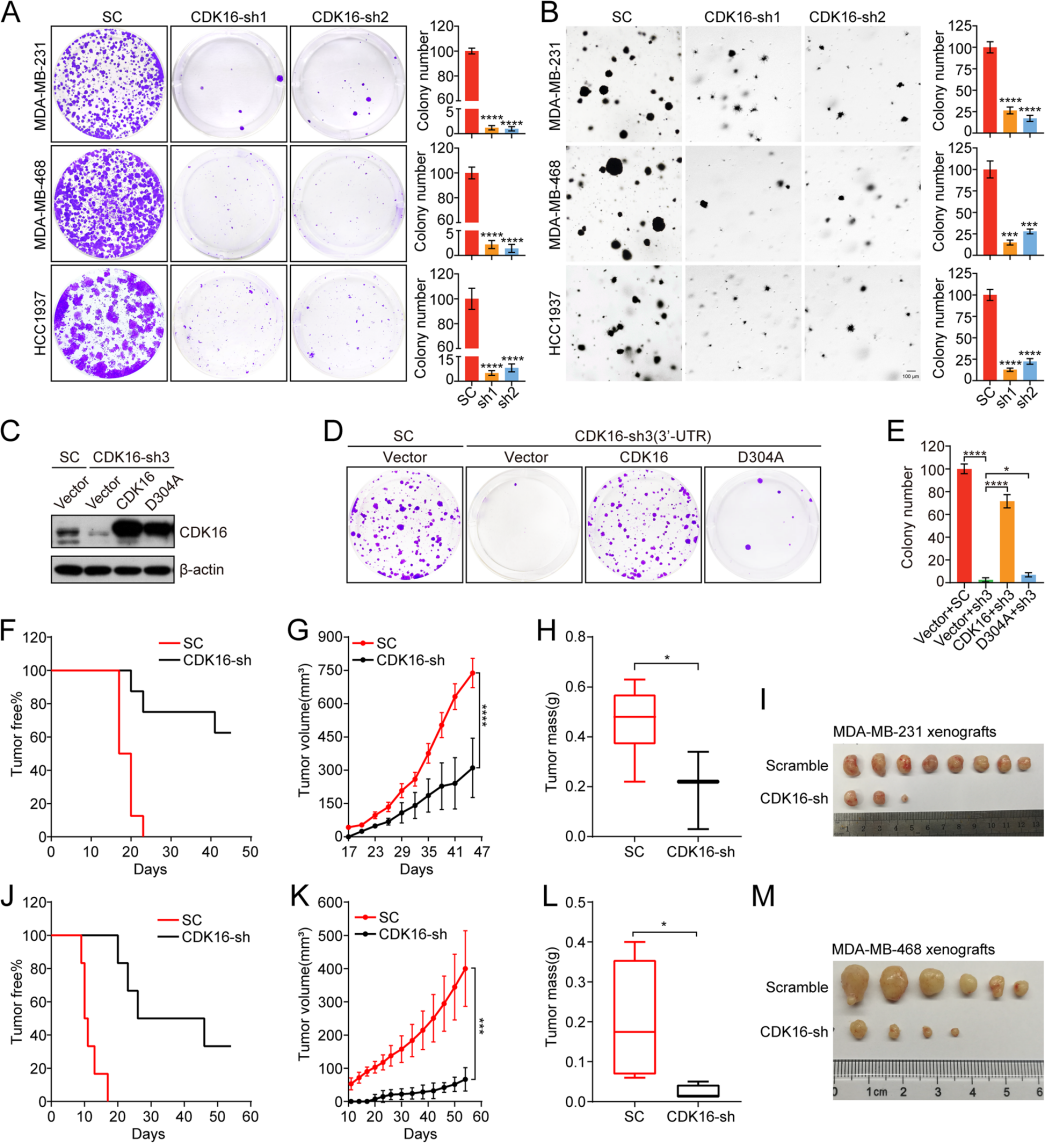
Knockdown of *CDK16* inhibits tumor growth of TNBC in vitro and in vivo. **A** 2D-colony formation assay of indicated TNBC cells transfected with scramble-shRNA or *CDK16*-shRNAs. Shown are representative images (left panel) and quantification of colonies (right panel). **B** 3D-colony formation assay of indicated CDK16-KD TNBC cells. Shown are representative images (left panel, scale bar: 100 μm) and quantification of colonies (right panel). **C-E** 2D-colony formation assay of MDA-MB-231 cells stably expressing control vector (vector), wild-type *CDK16* (CDK16) or kinase-inactive *CDK16*^*D304A*^ mutant (D304A) followed with transfection of scramble-shRNA or shRNA targeting the 3′-UTR of *CDK16*. Shown are immunoblot analysis for CDK16 expression (**C**), representative colony images (**D**), and quantification of colonies (**E**). **F-I** Tumorigenesis assay of MDA-MB-231 cells with CDK16-KD (*n* = 4 mice per group). Shown are tumor-free survival curve (**F**), tumor growth curve (**G**), statistics of tumor weight (**H**), and image of tumors (**I**). **J-M** Tumorigenesis assay of MDA-MB-468 cells with CDK16-KD (*n* = 3 mice per group). Shown are tumor-free survival curve (**J**), tumor growth curve (**K**), statistics of tumor weight (**L**), and image of tumors (**M**). Data are presented as mean ± SD (**A, B**, and **E**), mean ± SEM (**G** and **K**) or boxplot (**H** and **L**). *p* values were obtained by repeated measures two-way ANOVA (**G** and **K**), or two-tailed Student’s *t*-test (others). All *p < 0.05, ** *p* < 0.01, ***p < 0.001, **** *p* < 0.0001, ns, not significant

In vitro, 2D- and 3D-colony formation analyses showed that *CDK16* knockdown significantly reduced cell proliferation and clonogenic capacity of TNBC cells (Fig. 2A, B). In parallel, we observed that *CDK16* knockdown induced apparent growth defects in HR^+^ breast cancer cells but had only a slight effect on the proliferation of HER2^+^ cells (Fig. S2B, C). Flow cytometry analysis further showed that *CDK16* knockdown resulted in TNBC cell apoptosis (Fig. S2D) and G2/M cell cycle arrest (Fig. S2E).

To address whether the function of CDK16 depends on its enzymatic activity, we depleted endogenous *CDK16* by shRNA targeting 3′-UTR of *CDK16* in MDA-MB-231 cells, then restored shRNA-resistant wild-type *CDK16* (CDK16) or kinase-inactive *CDK16*^*D304A*^ mutant (D304A) [27]. Despite comparable overexpression efficiency (Fig. 2C), the wild-type *CDK16* but not the *CDK16*^*D304A*^ mutant could rescue cell proliferation defects shown by 2D-colony formation analysis (Fig. 2D, E), suggesting that the kinase activity is required for CDK16 function in TNBC.

In vivo, two cell line-derived xenograft (CDX) models were used to study the role of CDK16 in TNBC progression. MDA-MB-231 cells with CDK16-KD were orthotopically implanted into the mammary fat pads of nude recipients, and MDA-MB-468 cells with CDK16-KD were subcutaneously inoculated into the same strain recipients for tumorigenesis assay. *CDK16* knockdown dramatically delayed tumor formation (Fig. 2F) and suppressed tumor growth in the MDA-MB-231 tumor xenograft model, as indicated by reduced tumor volume, mass, and size in the CDK16-KD group compared to that in the control group (Fig. 2G-I). Consistent results were obtained from the MDA-MB-468 xenograft model (Fig. 2J-M). Together, these results demonstrated that CDK16 is critical for TNBC progression.

### Knockdown of CDK16 suppresses tumor progression of TNBC in patient-derived organoid and xenograft models

**Fig. 3:**
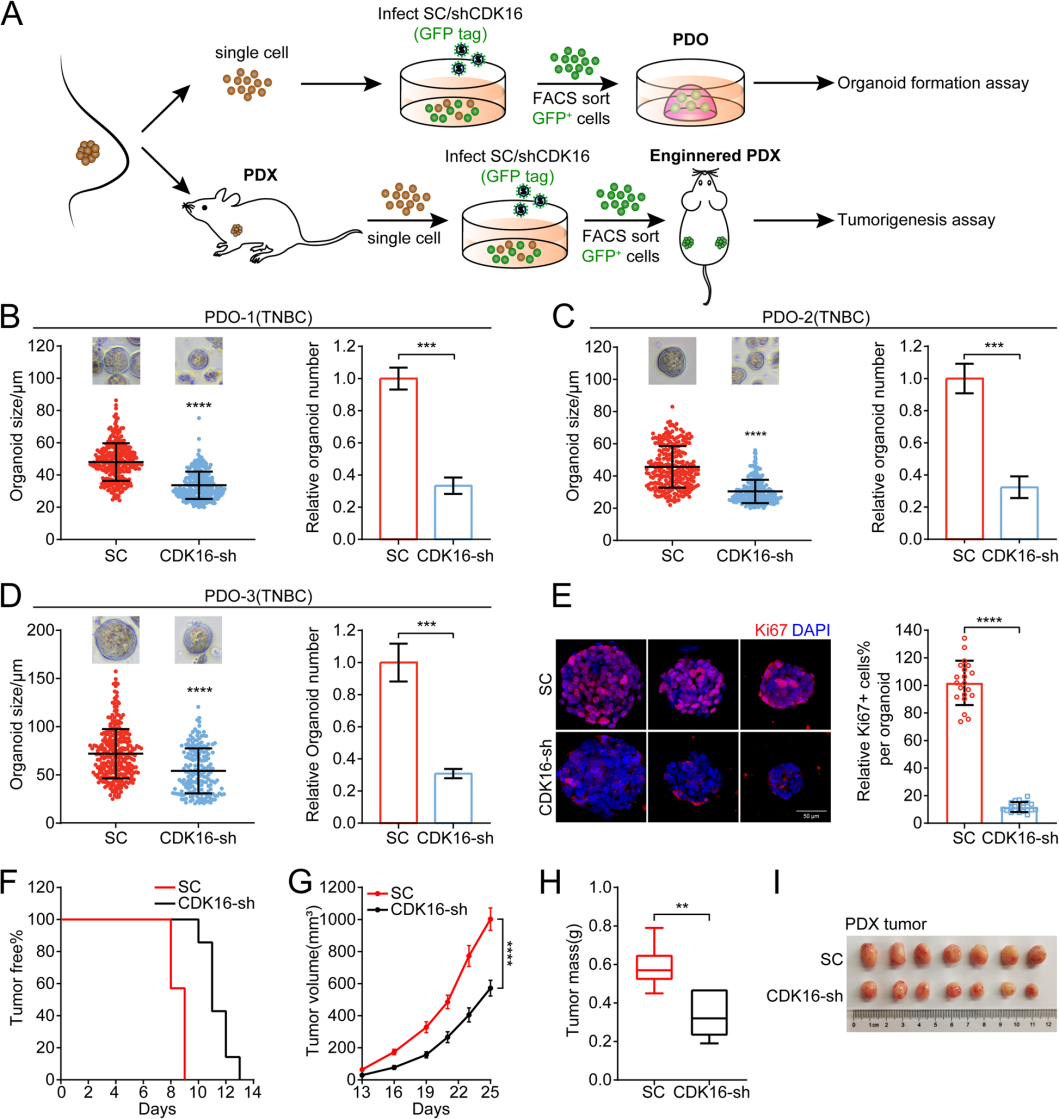
Knockdown of *CDK16* suppresses tumor progression of TNBC in patient-derived organoid and xenograft models. **A** Illustration of the establishment of PDO and PDX with CDK16-KD. **B-D** Organoid formation assay for three independent TNBC PDO models when *CDK16* was knocked down. Shown are statistics of tumor organoid size (left) and relative number of formed organoids (right). **E** Immunostaining for Ki67 (red) and DAPI (blue) of control and CDK16-KD organoids in PDO-3. Shown are representative images (left, scale bar: 50 μm) and a bar graph of the proportion of Ki67^+^ cells per organoid (right). **F-I** PDX lines stably transfected with scramble-shRNA or *CDK16*-shRNA were orthotopically transplanted into the mammary fat pads of nude mice (*n* = 4 mice per group) for tumorigenesis assay. Shown are tumor-free survival curve (**F**), tumor growth curve (**G**), statistics of tumor weight (**H**), and image of tumors (**I**). Data are presented as mean ± SD (**B, C, D,** and **E**), mean ± SEM (**G**), or boxplot (**H**). *p* values were obtained by repeated measures two-way ANOVA (**G**) or two-tailed Student’s *t*-test (others). All *p < 0.05, ** *p* < 0.01, ***p < 0.001, **** *p* < 0.0001, ns, not significant

In view of clinical implication, we generated PDO and PDX models to further assess the therapeutic benefits of targeting *CDK16* in TNBC. The tumor cells of three TNBC patients were dissociated and infected with scramble or shCDK16 virus carrying GFP tags, then GFP^+^ cells were isolated by FACS and cultured in a 3D system to generate tumor organoids. The tumor fragments from one patient were inoculated into SCID-Beige recipients, and a stable PDX line was successfully established. Tumor cells were isolated, infected, sorted from PDX tumors, and xenografted into nude recipients for tumorigenesis analysis (Fig. 3A). The knockdown efficiency of *CDK16* in the PDO and PDX models was confirmed by qPCR analysis (Fig. S3A).

Consistent with observations in CDX models, in all three PDO models, the organoid formation efficiency and organoid growth in the CDK16-KD group were significantly decreased compared with the control group, as shown by the reduction of size and number of organoids (Fig. 3B-D), indicating that *CDK16* knockdown suppresses TNBC progression. Additionally, Ki67 staining analysis of organoids showed that the proportion of Ki67^+^ cells in each organoid in the CDK16-KD group was markedly lower than that in the control group, corresponding to the proliferation-inhibited phenotype in CDK16-KD organoids (Fig. 3E). The results of the PDX model further verified the anti-tumor effect of targeting *CDK16* in TNBC, which significantly delayed tumor occurrence (Fig. 3F), suppressed tumor growth (Fig. 3G), and finally inhibited tumor progression (Fig. 3H, I).

We expanded the trial to include the luminal breast cancer subtype in the PDO model. The inhibitory effect of *CDK16* knockdown on the growth of organoids was also observed (Fig. S3B), although it was not as significant as that in TNBC. Taken together, these data suggested that targeting *CDK16* has great potential in the clinical treatment of TNBC.

### CDK16 promotes TNBC cell migration and tumor metastasis

**Fig. 4:**
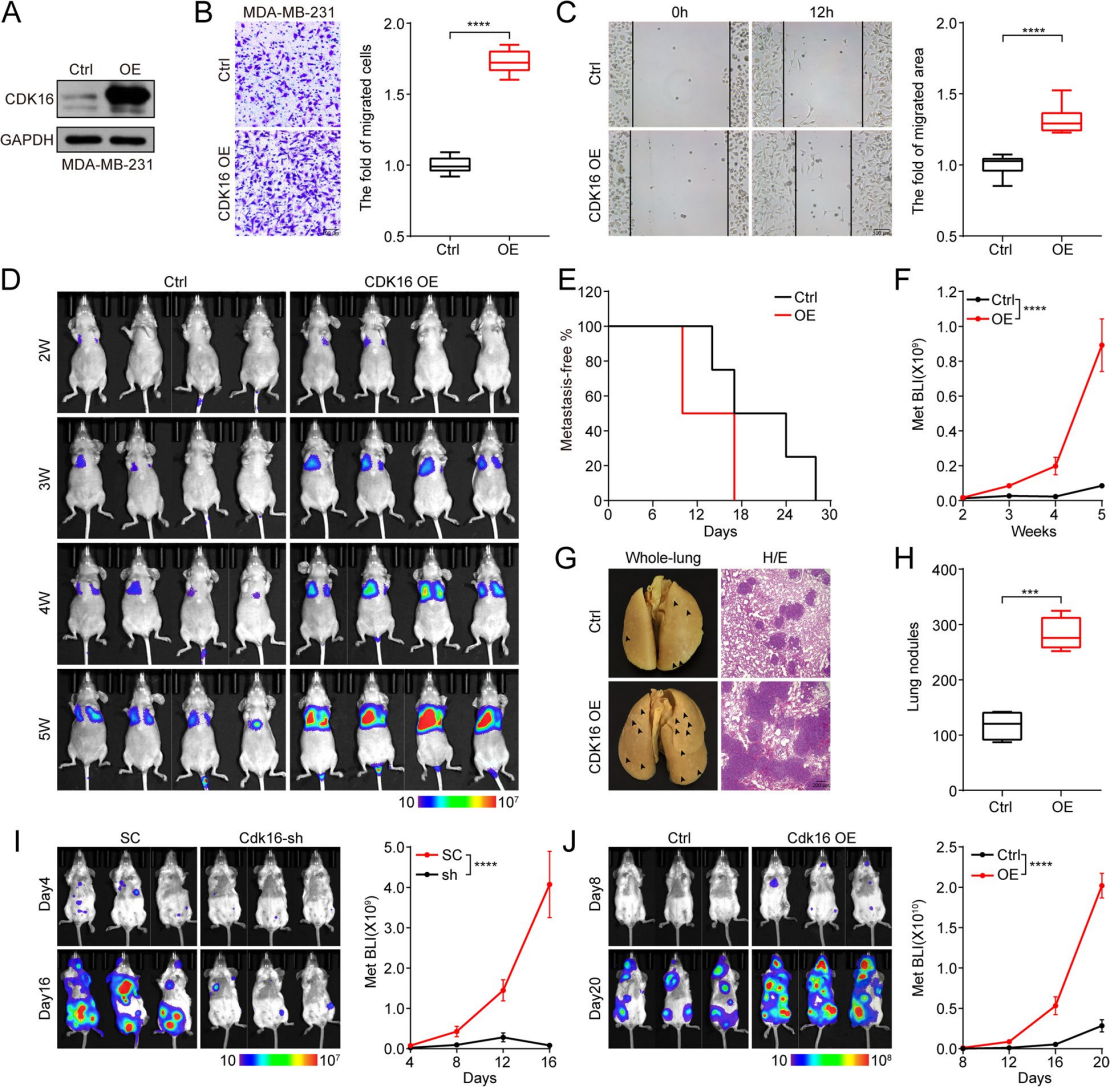
CDK16 promotes TNBC cell migration and tumor metastasis. **A-C** Immunoblot analysis to verify the overexpression of CDK16 (**A**), trans-well invasion assay (**B**, scale bar: 100 μm) and wound-healing assay (**C**, scale bar: 100 μm) of CDK16-OE MDA-MB-231 cells. **D-H** Intravenous injection of MDA-MB-231 stable lines transfected with control or *CDK16*-expressing vector into nude mice for lung metastasis analysis (*n* = 4 mice per group). Shown are BLI images (**D**), metastasis-free survival curve (**E**), quantification of lung metastasis by BLI (**F**), representative images of metastasized whole-lungs (left, arrows indicate lung nodules) and H/E staining of metastasized lung sections (right, scale bar: 200 μm) (**G**), and statistics of nodules on metastasized lung surface (**H**). **I-J** Left cardiac ventricle injection of 4 T1 stable lines transfected with scramble-shRNA or *CDK16*-shRNA (**I**), and EMT6 stable lines transfected with control or *CDK16*-overexpressing vector (**J**) into BALB/c mice for systemic metastasis analysis (*n* = 3 mice per group). Shown are BLI images (left) and quantification of whole-body metastasis by BLI (right). Data are presented as boxplot (**B, C,** and **H**) or mean ± SEM (**F, I,** and **J**). *p* values were obtained by two-tailed Student’s *t*-test (**B, C,** and **H**) or repeated measures two-way ANOVA (**F, I,** and **J**). All *p < 0.05, ** *p* < 0.01, ***p < 0.001, **** *p* < 0.0001, ns, not significant

The expression of CDK16 is positively correlated with the risk of metastasis and relapse in breast cancer patients, suggesting that CDK16 may contribute to TNBC metastasis. To test this possibility, we constructed an MDA-MB-231 stable line overexpressing *CDK16* (CDK16-OE), and labeled it with a luciferase reporter gene to quantitatively monitor tumor metastasis in vivo by bioluminescence imaging (BLI). The overexpression of CDK16 was confirmed by western blot analysis (Fig. 4A).

In vitro, trans-well and wound-healing assays showed that overexpression of *CDK16* significantly promoted cell invasion and migration (Fig. 4B, C). In vivo, we observed that the metastasis-free survival duration of mice bearing CDK16-OE cells was markedly shortened compared to that of mice bearing control cells (Fig. 4D, E), and overexpression of *CDK16* seriously aggravated lung-metastatic burden, as shown by BLI (Fig. 4D, F) and lung metastatic nodules (Fig. 4G, H). In addition, H/E analysis showed that overexpression of *CDK16* resulted in severe structural damage to lung tissues (Fig. 4G).

To further determine the role of CDK16 in TNBC metastasis, we knocked down *Cdk16* in high metastatic 4T1 cells and over-expressed *Cdk16* in relatively low metastatic EMT6 cells. The expression of Cdk16 was confirmed by western blot analysis (Fig. S4A, B). In vivo metastasis monitoring showed that knockdown of *Cdk16* strikingly reduced the distant organ metastasis of 4T1 cells (Fig. 4I), while over-expression of *Cdk16* significantly promoted distant metastasis of EMT6 cells to multiple organs demonstrated by in vivo and ex vivo BLI data (Fig. 4J and Fig. S4B). Together, these results demonstrated that CDK16 is essential for TNBC metastasis.

### CDK16 regulates cell proliferation by phosphorylating PRC1 and modulating spindle formation

**Fig. 5:**
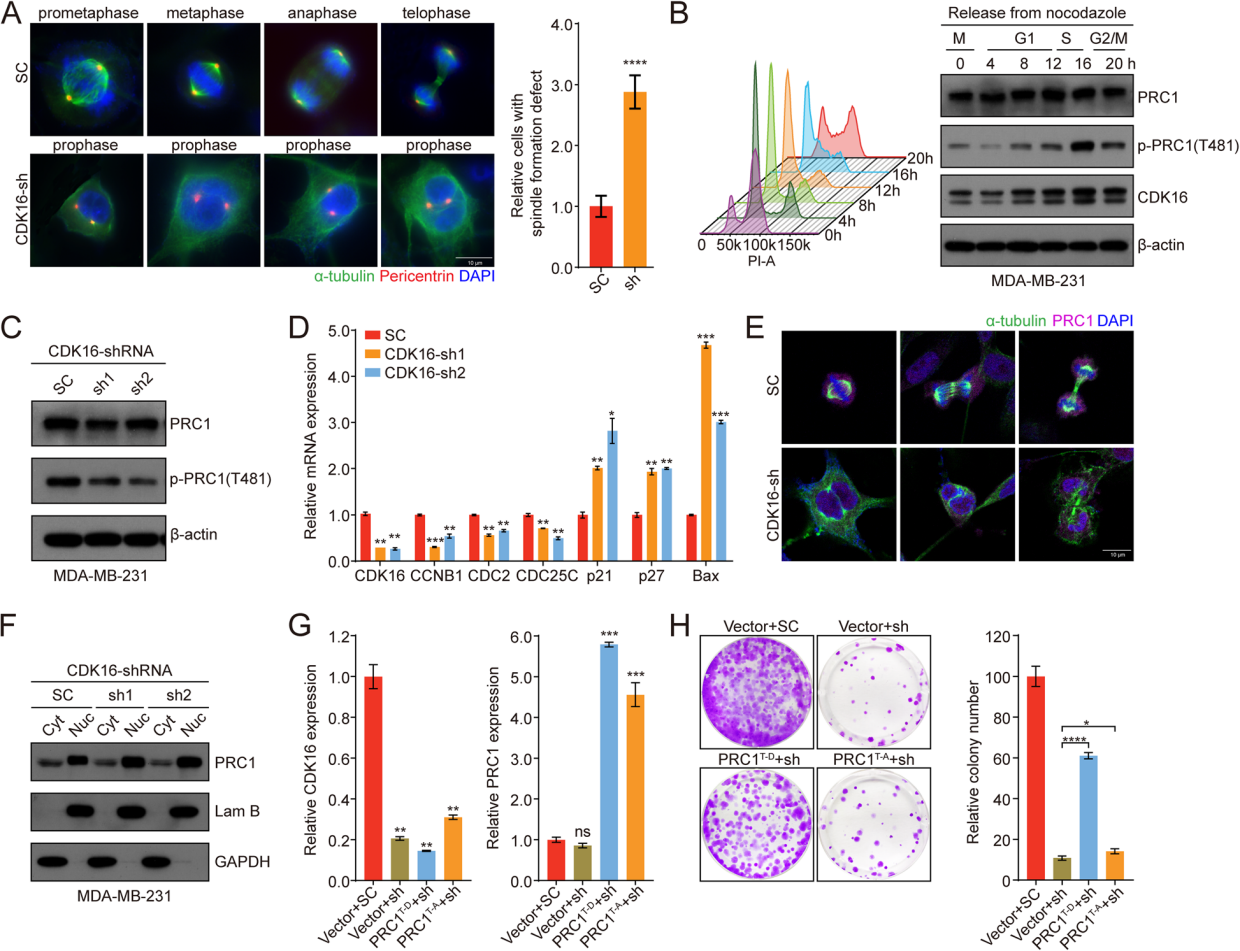
CDK16 regulates cell proliferation by phosphorylating PRC1 and modulating spindle formation. **A** Spindle formation analysis of control and CDK16-KD MDA-MB-231 cells. Cells were immunostained with antibodies for pericentrin (red), alpha-tubulin (green), followed by DAPI (blue) staining. Shown are representative immunofluorescence images of control cells in distinct cell cycle phases and CDK16-depleted cells stuck in prophase (left, scale bar: 10 μm), and statistics of cells with spindle formation defects (right). **B** MDA-MB-231 cells were synchronized in M phase by nocodazole followed by release back into the cell cycle, and the cell cycle phases were monitored by FACS (left). CDK16 and p-PRC1 (T481) expression was detected by immunoblot analysis at indicated time points (right). **C** Immunoblot analysis for total PRC1 and p-PRC1 (T481) expression of MDA-MB-231 cells when *CDK16* was knocked down. **D** qPCR analysis of PRC1-targeted cell cycle and apoptosis related genes in MDA-MB-231 cells when *CDK16* was knocked down. **E** Subcellular localization analysis of PRC1 in control and CDK16-KD MDA-MB-231 cells. Cells were immunostained with antibodies for PRC1 (purple), alpha-tubulin (green), followed by DAPI (blue) staining (scale bar: 10 μm). **F** Immunoblot analysis for PRC1 distribution in distinct subcellular fractions (Cyt: cytoplasm, Nuc: nucleus) of MDA-MB-231 cells when *CDK16* was knocked down. **G** qPCR analysis to verify the knockdown of *CDK16* and the overexpression of *PRC1*^*T481D*^ or *PRC1*^*T481A*^ mutant in MDA-MB-231 cells. **H** 2D-colony formation assay of MDA-MB-231 cells stably expressing control vector, *PRC1*^*T481D*^ or *PRC1*^*T481A*^ mutant followed with transfection of scramble-shRNA or *CDK16*-shRNA. Shown are representative images (left) and quantification of colonies (right). Data are presented as mean ± SD and *p* values were obtained by two-tailed Student’s *t*-test. All *p < 0.05, ** *p* < 0.01, ***p < 0.001, **** *p* < 0.0001, ns, not significant

To study the molecular mechanisms of CDK16 involved in TNBC progression, we profiled the transcriptome of CDK16-KD MDA-MB-231 cells with control cells by RNA sequencing. GO enrichment analysis showed that genes associated with neurogenesis [28], muscle [29], and exocytosis [30, 31] were down-regulated in CDK16-KD cells, consistent with the previously reported biological functions of CDK16. Interestingly, we observed that the microtubule cytoskeleton organization and spindle formation were also among the most affected processes (Fig. S5A). Therefore, we examined spindle formation during mitosis and observed apparent spindle formation defects in CDK16-KD MDA-MB-231 cells (Fig. 5A).

Microtubule-associated protein regulator of cytokinesis 1 (PRC1) is essential for spindle midzone formation and cytokinesis [32] and has been demonstrated as the substrate of several CDKs, such as CDK1, CDK2, and CDK16 [33, 34]. Intriguingly, TCGA analysis revealed that *PRC1* was highly expressed in breast cancer, especially in TNBC (Fig. S5B, C), and its high expression was significantly correlated with poor prognosis (Fig. S5D). Based on these findings, it is tempting to speculate that spindle formation defects elicited by *CDK16* knockdown may be mediated by PRC1. Indeed, immunoblot analysis showed that PRC1 phosphorylation at the T481 site (CDK-dependent major phosphorylation site) fluctuated with the abundance of CDK16 protein in the cell cycle process (Fig. 5B), and the phosphorylation level of PRC1 was clearly decreased upon *CDK16* knockdown in MDA-MB-231 cells (Fig. 5C). Furthermore, the expression of PRC1-targeted genes was significantly altered upon *CDK16* knockdown exemplified by the cell cycle-related genes (such as *CCNB1, CDC2,* and *CDC25C*) was down-regulated, and the apoptosis-related genes (such as *p21, p27,* and *Bax*) [35] was up-regulated (Fig. 5D). The subcellular localization of PRC1 determines its function in cell proliferation [36]. We further observed noticeable nuclear retention of PRC1 in CDK16-KD cells through immunostaining (Fig. 5E) and immunoblot analyses (Fig. 5F). The regulatory effect of CDK16 on PRC1 phosphorylation was further confirmed in PDO (Fig. S5E-G) and PDX (Fig. S5H) models.

Next, we investigated whether PRC1 could rescue cell proliferation defects induced by *CDK16* knockdown. 2D-proliferation analysis of MDA-MB-231 cells showed that the phosphor-mimic *PRC1*^*T481D*^ mutant significantly rescued the inhibited cell proliferation, whereas the phosphor-deficient *PRC1*^*T481A*^ mutant did not show the rescue effect (Fig. 5G, H), suggesting that the regulatory effect of CDK16 on cell proliferation is achieved through phosphorylation of PRC1 in TNBC. Collectively, these results indicated that CDK16 exerts its functions in TNBC by phosphorylating PRC1.

### Pharmacological inhibition of CDK16 suppresses tumor growth and metastasis of TNBC

**Fig. 6:**
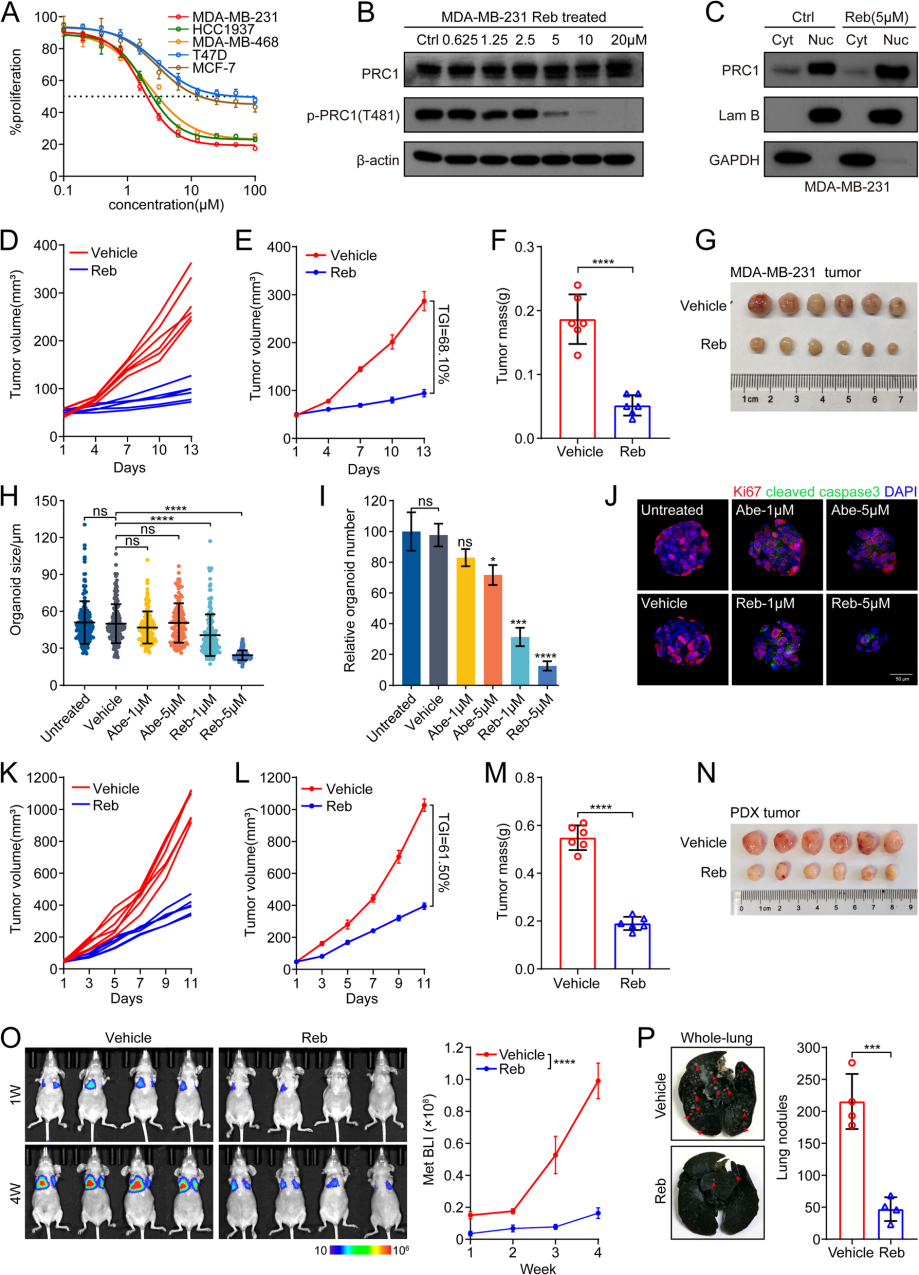
Pharmacological inhibition of CDK16 suppresses tumor growth and metastasis of TNBC. **A** MTT assay of indicated breast cancer cell lines upon increasing concentrations of Reb treatment for 72 h. **B** Immunoblot analysis of PRC1 and p-PRC1 expression in MDA-MB-231 cells treated with indicated concentrations of Reb for 48 h. **C** Immunoblot analysis for PRC1 distribution in distinct subcellular fractions (Cyt: cytoplasm, Nuc: nucleus) in MDA-MB-231 cells treated with Reb at dose of 5 μM for 48 h. **D-G** MDA-MB-231 cells were orthotopic xenografted into the mammary fat pads of nude recipients and mice bearing tumors were treated with vehicle or Reb (*n* = 3 mice per group). Shown are individual tumor growth curve (**D**), tumor average growth curve (**E**), statistics of tumor weight (**F**), and image of treated MDA-MB-231 tumors (**G**). **H-J** Organoid formation assay of a TNBC PDO model upon Reb or Abe treatment for 48 h. Shown are statistics of organoid size (**H**) and relative organoid number (**I**), IF analysis for Ki67 (red) and cleaved caspase-3 (green) followed by DAPI (blue) staining of organoids (**J**). **K-N** Nude mice bearing PDX tumors formed by orthotopic transplantation were treated with vehicle or Reb (*n* = 3 mice per group). Shown are individual tumor growth curve (**K**), tumor average growth curve (**L**), statistics of tumor weight (**M**), and image of treated PDX tumors (**N**). **O-P** Nude mice intravenously inoculated of MDA-MB-231 cells were treated with vehicle or Reb (*n* = 4 mice per group) for lung metastasis analysis. Shown are BLI images (left) and quantification of lung metastasis by BLI (right) (**O**), representative images of metastasized whole-lungs (left, arrows indicate lung nodules) and statistics of nodules on metastasized lung surface (right) (**P**). Data are presented as mean ± SEM (**E**, **L** and **O**) or mean ± SD (others). *p* values were obtained by repeated measures two-way ANOVA (**E**, **L** and **O**) or two-tailed Student’s *t*-test (others). All *p < 0.05, ** *p* < 0.01, ***p < 0.001, **** *p* < 0.0001, ns, not significant

Rebastinib (Reb) is a newly proposed inhibitor of CDK16, which can induce conformational changes in monomeric CDK16 and interfere its binding with cyclin Y, thereby inhibiting the catalytic activity of CDK16 [27]. To test whether TNBC can be treated with small molecular inhibitors targeting CDK16, we first examined the sensitivity of TNBC cell lines to Reb in vitro. MDA-MB-231, MDA-MB-468, and HCC1937 cells were treated with serial concentrations of Reb. MTT analysis showed that the viability of these TNBC cells was dramatically impaired by Reb treatment at micromolar concentrations (MDA-MB-231: IC50 = 1.726 μM; MDA-MB-468: IC50 = 2.191 μM; HCC1937: IC50 = 2.000 μM). Compared to TNBC cell lines, the luminal breast cancer cell lines MCF-7 and T47D were less sensitive (Fig. 6A). As expected, immunoblot analysis showed that Reb treatment reduced PRC1 phosphorylation at the T481 site and led to nuclear retention of PRC1 (Fig. 6B, C). In vivo, we assessed the anti-tumor efficiency of Reb using the xenograft model of MDA-MB-231 cells. Reb treatment markedly suppressed tumor growth (TGI = 68.10%), as shown by reduced individual tumor volume, average tumor volume, tumor weight, and tumor size (Fig. 6D-G).

To better assess the drug response of human primary tumors to Reb, the anti-tumor utility of Reb was further evaluated in TNBC PDO and PDX models. In the PDO model, both the size and number of tumor organoids were significantly decreased in the presence of Reb, but not in the presence of abemaciclib (Abe, CDK4/6 inhibitor, used as a control) (Fig. 6H, I). Immunostaining assessment of Ki67 and cleaved caspase-3 showed that Reb, but not Abe induced cell apoptosis and inhibited cell proliferation (Fig. 6J). In the PDX model, encouraging anti-tumor efficiency (TGI = 61.50%) of Reb was also observed (Fig. 6K-N).

We also tested the effect of Reb on TNBC metastasis using MDA-MB-231 lung metastasis mouse model. The results suggested that Reb administration effectively inhibited lung metastasis of MDA-MB-231 tumor cells, indicated by in vivo BLI data (Fig. 6O) and count of lung nodules (Fig. 6P).

Together, these results demonstrated that pharmacological inhibition of CDK16 effectively suppresses tumor growth and metastasis of TNBC, supporting CDK16 as a promising target for the treatment of TNBC.

### CDK16 inhibition is involved in multiple cancer-related signaling pathways

To understand the global biological effects caused by CDK16 inhibition, we conducted KEGG analysis of down-regulated genes in MDA-MB-231 cells with CDK16-KD and found that the signaling pathways related to cell proliferation and apoptosis, such as MAPK signaling and PI3K-Akt signaling pathways (Fig. S6A), changed significantly after *CDK16* knockdown. In addition, the results showed that CDK16 was involved in regulating the pluripotency of stem cells (Fig. S6A), suggesting the potential regulatory role of CDK16 in cancer stem cells (CSCs). Moreover, there are extensive interactions between theses signaling pathways (Fig. S6B). These evidences implicated that CDK16 may regulate the progression of TNBC through multiple mechanisms.

**Fig. 7:**
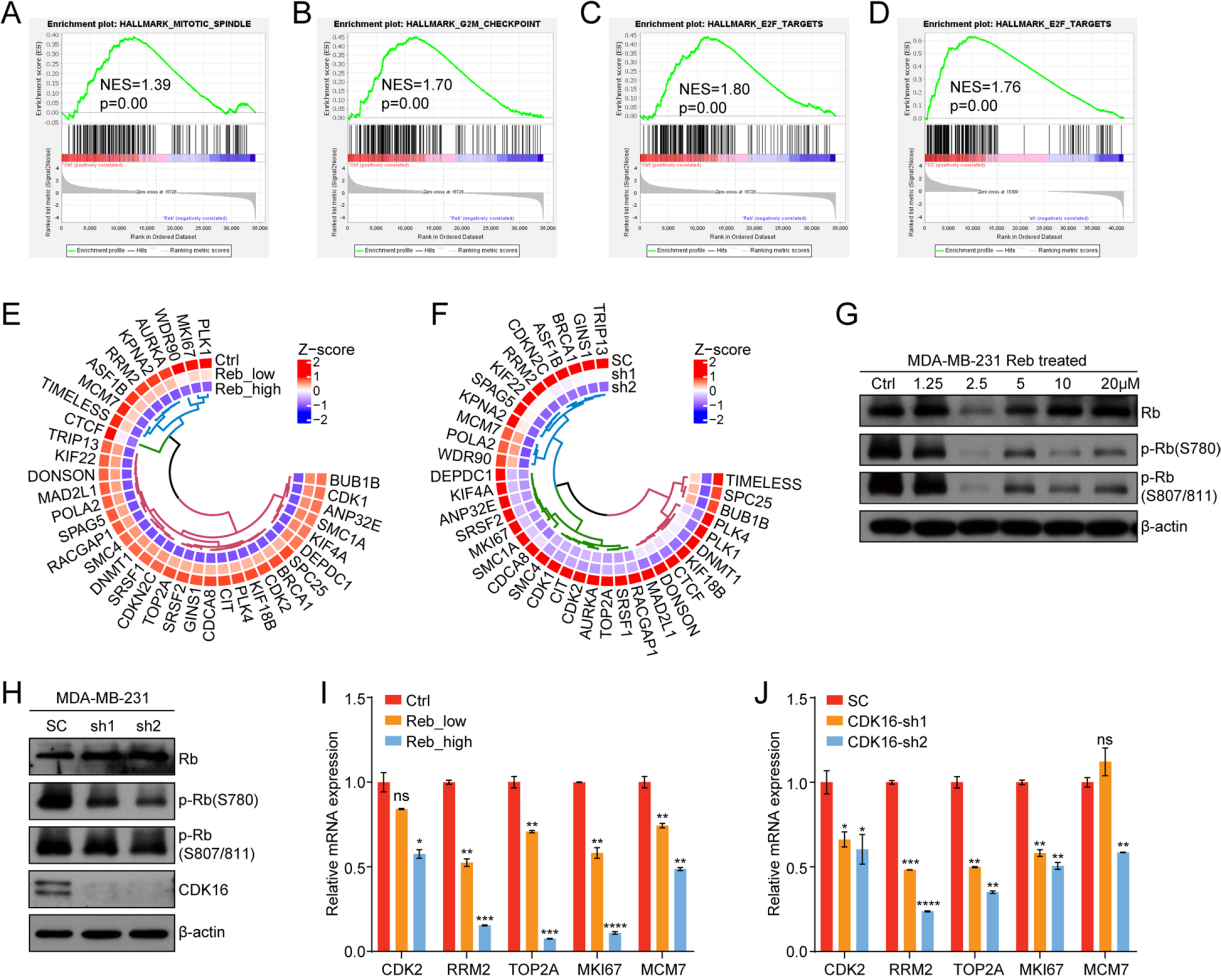
CDK16 inhibition is involved in multiple cancer-related signaling pathways. **A-B** GSEA analysis using RNA-seq data of MDA-MB-231 cells treated with low (1 μM) or high concentrations (5 μM) of Reb for 48 h. The GSEA plots showed mitotic spindle (**A**) and G2/M checkpoint signatures (**B**) were enriched in the control group. **C-D** GSEA analysis using RNA-seq data of MDA-MB-231 cells with Reb treatment (**C**) or with CDK16-KD (**D**). The GSEA plots showed E2F targets were enriched in the control groups. **E-F** Heat map shows the Z-score normalized expression of the most affected E2F target genes in MDA-MB-231 cells upon Reb treatment (**E**) or with CDK16-KD (**F**) according to RNA-seq data. The displayed genes are the intersection of the core enrichment genes from the GSEA analysis shown in (**C**) and (**D**). **G-H** Immunoblot analysis of total Rb and p-Rb expression in MDA-MB-231 cells treated with indicated concentrations of Reb for 48 h (**G**) or with CDK16-KD (**H**). **I-J** qPCR analysis of Rb-E2F direct targets expression in MDA-MB-231 cells treated with Reb_low (1 μM) or Reb_high (5 μM) for 48 h (**I**) or with CDK16-KD (**J**). Data are presented as mean ± SD and *p* values were obtained by two-tailed Student’s *t*-test (**I** and **J**). *p < 0.05, ** *p* < 0.01, ***p < 0.001, **** *p* < 0.0001, ns, not significant

Additionally, we performed transcriptomic analyses following Reb treatment in MDA-MB-231 cells. As expected, GSEA analysis revealed that gene signatures of mitotic spindle organization (Fig. 7A) and the G2/M checkpoint (Fig. 7B) were down-regulated in Reb-treated cells. Of note, the Rb-E2F gene signature was also impacted upon Reb treatment (Fig. 7C) or *CDK16* knockdown (Fig. 7D). The similar role of CDK4/6 in Rb-E2F pathway prompted us to deepen our understanding of the functions of CDK16. Heat map analysis showed that the target genes of the Rb-E2F pathway were down-regulated upon Reb treatment (Fig. 7E) or *CDK16* knockdown (Fig. 7F). Immunoblot analysis validated that Rb phosphorylation was significantly decreased upon Reb treatment (Fig. 7G) or *CDK16* knockdown (Fig. 7H). In addition, the expression of genes directly regulated by Rb-E2F signal (*CDK2*, *RRM2*, *TOP2A, MKI67*, and *MCM7*) was significantly down-regulated upon Reb treatment (Fig. 7I) or *CDK16* knockdown (Fig. 7J), as measured by qPCR analysis. Together, these results suggested that CDK16 regulates the Rb-E2F signaling by phosphorylating Rb.

Collectively, these data support that CDK16 inhibition is involved in multiple cancer-related signaling pathways, which may contribute to the function of CDK16 in regulating TNBC progression together with PRC1.

## Discussion

In this study, we demonstrated that CDK16 is a critical oncogenic driver in breast cancer. CDK16 is up-regulated in breast cancer with aberrantly high levels in TNBC, and elevated CDK16 expression is correlated with poor prognosis of breast cancer patients. Knockdown of *CDK16* significantly inhibits the proliferation of TNBC and HR^+^ breast cancer cells, but only has a slight effect on HER2^+^ breast cancer cells. Either genetic depletion or pharmacological inhibition of CDK16 effectively suppresses tumor growth and metastasis of TNBC in various tumor models. Mechanistically, CDK16 exerts its function by phosphorylating PRC1 at the T481 site and regulating spindle formation during mitosis. Inhibition of CDK16 induces spindle formation defects, subsequently leads to G2/M arrest and cell apoptosis. Furthermore, our results show that CDK16 has a similar role to CDK4/6 in phosphorylating Rb and regulating the Rb-E2F signaling pathway, which is essential for G1/S transition. Therefore, CDK16 inhibition interrupts the cell cycle at different phases, offering the possibility of CDK16 as a novel target for the treatment of TNBC.

The newly proposed atypical CDK members PFTAIRE1–2 (CDK14–15) and PCTAIRE1–3 (CDK16–18) kinases share CCNY as an activator and are co-expressed in various tissues with the highest expression in brain and testis [16]. The functions and regulatory networks of these atypical CDKs are largely unknown. Knocking down individual atypical CDKs failed to cause any phenotype, suggesting possible functional redundancy of these kinases [37]. However, a functional study of CDK16 using CDK16 conditional knockout mouse model has validated its essential role in spermatogenesis [38]. Here, our study demonstrated that CDK16 phosphorylates PRC1 and Rb, thereby regulating spindle formation and the Rb-E2F pathway, providing new insights into the function of the atypical CDK subfamily.

PRC1 is recognized as an oncoprotein in various cancer types, including breast cancer [39–42]. PRC1 deficiency leads to cell cycle G2/M arrest and apoptosis, consistent with a CDK16-inhibited phenotype. Our results demonstrate that CDK16 affects the subcellular localization of PRC1 and regulates mitotic spindle formation by phosphorylating the T481 site of PRC1. In early mitosis, phosphorylated PRC1 is localized to the mitotic spindle, and its bundling activity is strongly inhibited. Then PRC1 is dephosphorylated and translocated to the spindle midzone/midbody during anaphase and cytokinesis [36]. Here, we revealed that the PRC1 phospho-mimic PRC1^T481D^ mutant could partially rescue the cell proliferation defect induced by CDK16 deletion in TNBC cells. Our findings support that CDK16-mediated phosphorylation of PRC1 at the T481 site is required for cell cycle progression. It is important to point out although PRC1 is a potential therapeutic target for multiple cancer types, there are no known specific inhibitors directly targeting PRC1. Therefore, our study expands the understanding of PRC1 function and provides an alternative tumor therapy strategy: inhibiting PRC1 by targeting its regulatory kinase, CDK16.

Rebastinib is a preclinically tested compound that was first developed to overcome the clinically relevant CML drug-resistant BCR-ABL1^T315I^ mutant [43]. Recently, rebastinib has been identified as a potent inhibitor of CDK16. It is worth noting that rebastinib is a multi-target agent against other kinases like Tie2 [44]. It has been reported that in the MMTV-PyMT mouse model of breast cancer, twice-daily oral administration of 10 mg/kg rebastinib significantly reduced tumor growth and lung metastasis by blocking the recruitment and function of Tie2^Hi^ macrophages [45]. We also observed the additive effect of rebastinib on Tie2-expressing macrophages in our study (data not shown). Targeting CDK16 and Tie2 may jointly promote the anti-tumor activity of rebastinib in TNBC. Nevertheless, the compatible anti-tumor activity produced by the inhibition of Tie2 and CDK16 further establishes the value of rebastinib as an effective anti-tumor compound. Our study supports CDK16 as a potential therapeutic target for TNBC and provides evidence and guidance for developing CDK16 inhibitors as anti-tumor agents.

The CDK-Rb-E2F pathway is involved in various biological processes such as cell cycle, apoptosis, autophagy, angiogenesis, and epithelial-to-mesenchymal transition (EMT) [46]. Dysregulation of the CDK-Rb-E2F pathway has been found in almost all human malignancies, making it an attractive target for cancer therapy. The significance of the CyclinD-CDK4/6-Rb-E2F pathway in controlling G1/S transition during cell cycle progression has led to the development of CDK4/6 inhibitors for clinical cancer therapy [13]. To date, CDK4/6 inhibitors have been approved for the treatment of advanced HR^+^/HER2^−^ breast cancer, whereas TNBC tumors are relatively resistant to CDK4/6 inhibitors [47]. Here, we found that CDK16 also phosphorylates Rb, and both TNBC and HR^+^ breast cancer cells are sensitive to CDK16 inhibition. Importantly, CDK16 inhibition has a comparable inhibitory effect on the proliferation of Rb-deficient MDA-MB-468 and Rb-proficient MDA-MB-231 cell lines, suggesting that CDK16 exerts its function through multiple mechanisms, not only regulating the Rb-E2F pathway.

CCNY is reported to regulate the proliferation, migration, and invasion of cancer cells through the Wnt/β-catenin signaling pathway [17]. Of note, independent studies have revealed that PRC1 is involved in tumorigenesis via the Wnt/β-catenin signaling pathway [35, 40]. Accordingly, we reasoned that CDK16, CCNY, and PRC1 jointly regulate TNBC progression through the Wnt/β-catenin signaling pathway. However, we did not detect a prominent regulatory effect of CDK16 on the Wnt signaling pathway in our study (data not shown). This may be because PRC1 phosphorylation mediated by CDK16 is critical for spindle formation but not for Wnt signaling transduction. In addition to PRC1, other pathways of cell proliferation and apoptosis, such as MAPK signal and PI3K-Akt signaling pathways, may also contribute to the function of CDK16 in regulating TNBC progression. Considering that CCNY is a shared cyclin partner of atypical CDKs, CCNY may regulate Wnt signaling by phosphorylating LRP5/6 together with CDK14 (PFTK1) [48], which further reflects the complexity of cyclin-CDK function.

## Conclusions

In this study, we investigated the clinical relevance of CDK16 in the prognosis of breast cancer patients, and revealed that CDK16 is highly expressed in breast cancers, especially TNBC, and its elevated expression is correlated with poor prognosis. We further studied the function and underlying molecular mechanisms of CDK16 in TNBC. In vitro, *CDK16* deficiency markedly inhibited the proliferation and migration of TNBC cells. In vivo, in various tumor models, CDK16 deficiency significantly suppressed tumor growth and metastasis of TNBC, while CDK16 overexpression remarkably promoted metastasis of TNBC. The pharmacological inhibition of rebastinib on CDK16 also achieved encouraging anti-tumor efficiency in TNBC. In terms of the mechanisms, CDK16 plays its oncogenic role by phosphorylating PRC1 and regulating spindle formation during mitosis. Interestingly, CDK16 inhibition also inhibits Rb phosphorylation and Rb-E2F signal. In conclusion, our study provides new insights into the roles of atypical CDKs in cancer and emphasizes that CDK16 is a promising therapeutic target for TNBC.

## Supplementary Information

**Additional file 1: Supplementary Figure S1.** Clinical relevance analysis of atypical CDKs (*CDK14, 15, 17, 18*) in breast cancer. A mRNA expression analysis of *CDK14* in different subtypes of breast cancer using data from TCGA breast cancer dataset. B Survival analysis of overall survival (OS), recurrence-free survival (RFS), and distant metastasis-free survival (DMFS) for breast cancer patients with stratified mRNA expression of *CDK14* by KM plotter. C mRNA expression analysis of *CDK15* in different subtypes of breast cancer using data from TCGA breast cancer dataset. D Survival analysis of OS, RFS, and DMFS for breast cancer patients with stratified mRNA expression of *CDK15* by KM plotter. E mRNA expression analysis of *CDK17* in different subtypes of breast cancer using data from TCGA breast cancer dataset. F Survival analysis of OS, RFS, and DMFS for breast cancer patients with stratified mRNA expression of *CDK17* by KM plotter. G mRNA expression analysis of *CDK18* in different subtypes of breast cancer using data from TCGA breast cancer dataset. H Survival analysis of OS, RFS, and DMFS for breast cancer patients with stratified mRNA expression of *CDK18* by KM plotter. Data are presented as mean ± SD (A, C, E, and G). *p* values were obtained by two-tailed Student’s *t*-test (A, C, E, and G) or log rank test (others). All *p < 0.05, ** *p* < 0.01, ***p < 0.001, **** *p* < 0.0001, ns, not significant.

**Additional file 2: Supplementary Figure S2.** *CDK16* knockdown induces proliferation inhibition in TNBC and HR^+^ cells but not in HER2^+^ breast cancer cells. A-B Immunoblot analysis to verify CDK16 knockdown in indicated TNBC cells (A) and in HR^+^ and HER2^+^ breast cancer cells (B) transfected with two individual *CDK16*-shRNAs. C 2D-colony formation assay of indicated HR^+^ and HER2^+^ cells with CDK16-KD. Shown are representative images (scale bar, 100 μm, left panel) and quantification of colonies (right panel). D Apoptosis analysis of indicated TNBC cells with CDK16-KD. Shown are FACS results (left panel) and statistics of cells in early apoptosis (right panel). E Cell cycle analysis of indicated TNBC cells with CDK16-KD. Shown are FACS results (left panel) and statistics of cells in specific phases (right panel). Data are shown as mean ± SD and *p* values were obtained by two-tailed Student’s *t*-test. All *p < 0.05, ** *p* < 0.01, ***p < 0.001, **** *p* < 0.0001, ns, not significant.

**Additional file 3: Supplementary Figure S3.** Knockdown efficiency of *CDK16* in PDO and PDX models. A qPCR analysis to verify *CDK16* knockdown in three independent TNBC PDOs and the PDX model. B Organoid formation assay for the established LumB PDO model when *CDK16* was knocked down. Shown are qPCR analysis to verify *CDK16* knockdown (left), statistics of tumor organoid size (middle), and relative number of formed organoids (right). Data are presented as mean ± SD and *p* values were obtained by two-tailed Student’s *t*-test. All *p < 0.05, ** *p* < 0.01, ***p < 0.001, **** *p* < 0.0001, ns, not significant.

**Additional file 4: Supplementary Figure S4.** Cdk16 promotes tumor metastasis of mouse TNBC in systemic metastasis models. A Immunoblot analysis to verify Cdk16 knockdown in 4T1 cells and Cdk16 overexpression in EMT6 cells. B BLI images of major organs dissected from mice bearing control or Cdk16-OE EMT6 cells showed the metastatic sites of tumor cells.

**Additional file 5: Supplementary Figure S5.** CDK16 regulates tumor growth of TNBC by phosphorylating PRC1. A GO analysis of down-regulated genes in CDK16-KD MDA-MB-231 cells according to RNA-seq data. B-C PRC1 mRNA expression in normal and breast cancer tissues (B), and in different subtypes (C) of TCGA samples in the ULCAN database. D Survival analysis of OS, RFS, and DMFS for breast cancer patients with low or high mRNA expression of *PRC1* by KM plotter. E-F IF analysis for PRC1 and p-PRC1(T481) of control and CDK16-KD organoids in established PDO model. Shown are representative IF images (E) and statistics of the proportion of p-PRC1(T481) positive cells per organoid (F). G Immunoblot analysis for PRC1 and p-PRC1(T481) expression in control and CDK16-KD organoids formed in (E). H IF analysis of control and CDK16-KD PDX tumor sections for PRC1 (left panel) and p-PRC1(T481) (right panel) followed by DAPI staining. Data are presented as mean ± SD (B, C, and F). *p* values were obtained by unpaired two-tailed *t* test (B, C, and F) or log rank test (D). All *p < 0.05, ** *p* < 0.01, ***p < 0.001, **** *p* < 0.0001, ns, not significant.

**Additional file 6: Supplementary Figure S6.** CDK16 inhibition is involved in multiple cancer-related signaling pathways. A KEGG analysis of the significantly decreased pathways in CDK16-KD MDA-MB-231 cells. B Network and key genes of the representative pathways enriched in the KEGG analysis.

**Additional file 7: Supplementary Table S1.** List of primers used in qPCR analysis.

**Additional file 8: Supplementary Table S2.** List of the antibodies used and their application.

## Data Availability

RNA-seq data generated in this study are publicly available in GEO dataset under accession number GSE189758.

## Abbreviations

CDK: Cyclin-Dependent Kinase
CCNY: Cyclin Y
CCNYL1: Cyclin Y-like 1
PRC1: Protein Regulator of Cytokinesis 1
TNBC: Triple-Negative Breast Cancer
CML: Chronic Myelogenous Leukemia
SERM: Selective Estrogen Receptor Modulator
SERD: Selective Estrogen Receptor Degrader
FACS: Fluorescence-activated Cell Sorting
IF: Immunofluorescence
IHC: Immunohistochemistry
HE: Hematoxylin-eosin
BLI: Bioluminescence Imaging
UTR: Untranslated region
CDX: Cell line-derived xenograft
PDX: Patient-derived xenograft
PDO: Patient-derived organoid
TCGA: The Cancer Genome Atlas
GEO: Gene Expression Omnibus
CPTAC: Clinical Proteomic Tumor Analysis Consortium
KM-plotter: Kaplan-Meier plotter
GO: Gene Ontology
KEGG: Kyoto Encyclopedia of Genes and Genomes
GSEA: Gene Set Enrichment Analysis
EMT: Epithelial-to-Mesenchymal transition
RNA-seq: RNA sequencing

## References

[1] Siegel RL, Miller KD, Jemal A (2020). Cancer statistics, 2020. CA Cancer J Clin.

[2] Goldhirsch A, Wood WC, Coates AS, Gelber RD, Thürlimann B, Senn HJ (2011). Strategies for subtypes--dealing with the diversity of breast cancer: highlights of the St. Gallen international expert consensus on the primary therapy of early breast Cancer 2011. Ann Oncol.

[3] Dent R, Trudeau M, Pritchard KI, Hanna WM, Kahn HK, Sawka CA (2007). Triple-negative breast cancer: clinical features and patterns of recurrence. Clin Cancer Res.

[4] Burstein HJ, Temin S, Anderson H, Buchholz TA, Davidson NE, Gelmon KE (2014). Adjuvant endocrine therapy for women with hormone receptor-positive breast cancer: american society of clinical oncology clinical practice guideline focused update. J Clin Oncol.

[5] Ramakrishna N, Temin S, Chandarlapaty S, Crews JR, Davidson NE, Esteva FJ (2014). Recommendations on disease management for patients with advanced human epidermal growth factor receptor 2-positive breast cancer and brain metastases: American Society of Clinical Oncology clinical practice guideline. J Clin Oncol.

[6] Metzger-Filho O, Tutt A, de Azambuja E, Saini KS, Viale G, Loi S (2012). Dissecting the heterogeneity of triple-negative breast cancer. J Clin Oncol.

[7] Telli ML, Timms KM, Reid J, Hennessy B, Mills GB, Jensen KC (2016). Homologous recombination deficiency (HRD) score predicts response to platinum-containing Neoadjuvant chemotherapy in patients with triple-negative breast Cancer. Clin Cancer Res.

[8] Morgan DO (1995). Principles of CDK regulation. Nature..

[9] Malumbres M, Barbacid M (2005). Mammalian cyclin-dependent kinases. Trends Biochem Sci.

[10] Malumbres M, Harlow E, Hunt T, Hunter T, Lahti JM, Manning G (2009). Cyclin-dependent kinases: a family portrait. Nat Cell Biol.

[11] Malumbres M (2014). Cyclin-dependent kinases. Genome Biol.

[12] Hanahan D, Weinberg RA (2011). Hallmarks of cancer: the next generation. Cell..

[13] Scott SC, Lee SS, Abraham J (2017). Mechanisms of therapeutic CDK4/6 inhibition in breast cancer. Semin Oncol.

[14] Wang Y, Zhang T, Kwiatkowski N, Abraham BJ, Lee TI, Xie S (2015). CDK7-dependent transcriptional addiction in triple-negative breast cancer. Cell..

[15] Quereda V, Bayle S, Vena F, Frydman SM, Monastyrskyi A, Roush WR (2019). Therapeutic targeting of CDK12/CDK13 in triple-negative breast Cancer. Cancer Cell.

[16] Mikolcevic P, Rainer J, Geley S (2012). Orphan kinases turn eccentric: a new class of cyclin Y-activated, membrane-targeted CDKs. Cell Cycle.

[17] Liu H, Shi H, Fan Q, Sun X (2016). Cyclin Y regulates the proliferation, migration, and invasion of ovarian cancer cells via Wnt signaling pathway. Tumour Biol.

[18] Yue W, Zhao X, Zhang L, Xu S, Liu Z, Ma L (2011). Cell cycle protein cyclin Y is associated with human non-small-cell lung cancer proliferation and tumorigenesis. Clin Lung Cancer.

[19] Xu YG, Zhi W, Jie W, Li JH, Wu Y (2010). Lentivirus-mediated knockdown of cyclin Y (CCNY) inhibits glioma cell proliferation. Oncol Res.

[20] Yan F, Wang X, Zhu M, Hu X (2016). RNAi-mediated downregulation of cyclinY to attenuate human breast cancer cell growth. Oncol Rep.

[21] Si Y, Liu J, Shen H, Zhang C, Wu Y, Huang Y (2019). Fisetin decreases TET1 activity and CCNY/CDK16 promoter 5hmC levels to inhibit the proliferation and invasion of renal cancer stem cell. J Cell Mol Med.

[22] Zeng L, Cai C, Li S, Wang W, Li Y, Chen J (2016). Essential roles of Cyclin Y-like 1 and Cyclin Y in dividing Wnt-responsive mammary stem/progenitor cells. PLoS Genet.

[23] DeRose YS, Gligorich KM, Wang G, Georgelas A, Bowman P, Courdy SJ (2013). Patient-derived models of human breast cancer: protocols for in vitro and in vivo applications in tumor biology and translational medicine. Curr Protoc Pharmacol.

[24] Rueda OM, Sammut SJ, Seoane JA, Chin SF, Caswell-Jin JL, Callari M (2019). Dynamics of breast-cancer relapse reveal late-recurring ER-positive genomic subgroups. Nature..

[25] Pereira B, Chin SF, Rueda OM, Vollan HK, Provenzano E, Bardwell HA (2016). The somatic mutation profiles of 2,433 breast cancers refines their genomic and transcriptomic landscapes. Nat Commun.

[26] Curtis C, Shah SP, Chin SF, Turashvili G, Rueda OM, Dunning MJ (2012). The genomic and transcriptomic architecture of 2,000 breast tumours reveals novel subgroups. Nature..

[27] Dixon-Clarke SE, Shehata SN, Krojer T, Sharpe TD, von Delft F, Sakamoto K (2017). Structure and inhibitor specificity of the PCTAIRE-family kinase CDK16. Biochem J.

[28] Graeser R, Gannon J, Poon R, Dubois T, Hunt T (2002). Regulation of the CDK-related protein kinase PCTAIRE-1 and its possible role in neurite outgrowth in Neuro-2A cells. J Cell Sci.

[29] Shimizu K, Uematsu A, Imai Y, Sawasaki T (2014). Pctaire1/Cdk16 promotes skeletal myogenesis by inducing myoblast migration and fusion. FEBS Lett.

[30] Palmer KJ, Konkel JE, Stephens DJ (2005). PCTAIRE protein kinases interact directly with the COPII complex and modulate secretory cargo transport. J Cell Sci.

[31] Liu Y, Cheng K, Gong K, Fu AK, Ip NY (2006). Pctaire1 phosphorylates N-ethylmaleimide-sensitive fusion protein: implications in the regulation of its hexamerization and exocytosis. J Biol Chem.

[32] Mollinari C, Kleman JP, Jiang W, Schoehn G, Hunter T, Margolis RL (2002). PRC1 is a microtubule binding and bundling protein essential to maintain the mitotic spindle midzone. J Cell Biol.

[33] Jiang W, Jimenez G, Wells NJ, Hope TJ, Wahl GM, Hunter T (1998). PRC1: a human mitotic spindle-associated CDK substrate protein required for cytokinesis. Mol Cell.

[34] Hernández-Ortega S, Sánchez-Botet A, Quandt E, Masip N, Gasa L, Verde G (2019). Phosphoregulation of the oncogenic protein regulator of cytokinesis 1 (PRC1) by the atypical CDK16/CCNY complex. Exp Mol Med.

[35] Zhan P, Zhang B, Xi GM, Wu Y, Liu HB, Liu YF (2017). PRC1 contributes to tumorigenesis of lung adenocarcinoma in association with the Wnt/beta-catenin signaling pathway. Mol Cancer.

[36] Zhu C, Lau E, Schwarzenbacher R, Bossy-Wetzel E, Jiang W (2006). Spatiotemporal control of spindle midzone formation by PRC1 in human cells. Proc Natl Acad Sci U S A.

[37] Davidson G, Shen J, Huang YL, Su Y, Karaulanov E, Bartscherer K (2009). Cell cycle control of wnt receptor activation. Dev Cell.

[38] Mikol Ce Vic P, Sigl R, Rauch V, Hess MW, Pfaller K, Barisic M (2012). Cyclin-dependent kinase 16/PCTAIRE kinase 1 is activated by Cyclin Y and is essential for spermatogenesis. Mol Cell Biol.

[39] Wu F, Shi X, Zhang R, Tian Y, Wang X, Wei C (2018). Regulation of proliferation and cell cycle by protein regulator of cytokinesis 1 in oral squamous cell carcinoma. Cell Death Dis.

[40] Chen J, Rajasekaran M, Xia H, Zhang X, Kong SN, Sekar K (2016). The microtubule-associated protein PRC1 promotes early recurrence of hepatocellular carcinoma in association with the Wnt/beta-catenin signalling pathway. Gut..

[41] Liu X, Li Y, Meng L, Liu XY, Peng A, Chen Y (2018). Reducing protein regulator of cytokinesis 1 as a prospective therapy for hepatocellular carcinoma. Cell Death Dis.

[42] Shimo A, Nishidate T, Ohta T, Fukuda M, Nakamura Y, Katagiri T (2007). Elevated expression of protein regulator of cytokinesis 1, involved in the growth of breast cancer cells. Cancer Sci.

[43] Eide CA, Adrian LT, Tyner JW, Mac Partlin M, Anderson DJ, Wise SC (2011). The ABL switch control inhibitor DCC-2036 is active against the chronic myeloid leukemia mutant BCR-ABLT315I and exhibits a narrow resistance profile. Cancer Res.

[44] Koch PD, Ahmed MS, Kohler RH, Li R, Weissleder R (2020). Imaging of Tie2 with a fluorescently labeled small molecule affinity ligand. ACS Chem Biol.

[45] Harney AS, Karagiannis GS, Pignatelli J, Smith BD, Kadioglu E, Wise SC (2017). The selective Tie2 inhibitor Rebastinib blocks recruitment and function of Tie2(hi) macrophages in breast Cancer and pancreatic neuroendocrine tumors. Mol Cancer Ther.

[46] Johnson J, Thijssen B, McDermott U, Garnett M, Wessels LF, Bernards R (2016). Targeting the RB-E2F pathway in breast cancer. Oncogene..

[47] Finn RS, Dering J, Conklin D, Kalous O, Cohen DJ, Desai AJ (2009). PD 0332991, a selective cyclin D kinase 4/6 inhibitor, preferentially inhibits proliferation of luminal estrogen receptor-positive human breast cancer cell lines in vitro. Breast Cancer Res.

[48] Ou-Yang J, Huang LH, Sun XX (2017). Cyclin-dependent kinase 14 promotes cell proliferation, migration and invasion in ovarian Cancer by inhibiting Wnt signaling pathway. Gynecol Obstet Investig.

