# Supplementary figures and images for: CDK16 promotes the progression and metastasis of triple-negative breast cancer by phosphorylating PRC1
Source: J Exp Clin Cancer Res. 2022 Apr 21;41:149. doi: 10.1186/s13046-022-02362-w (PMC9027050; doi:10.1186/s13046-022-02362-w)

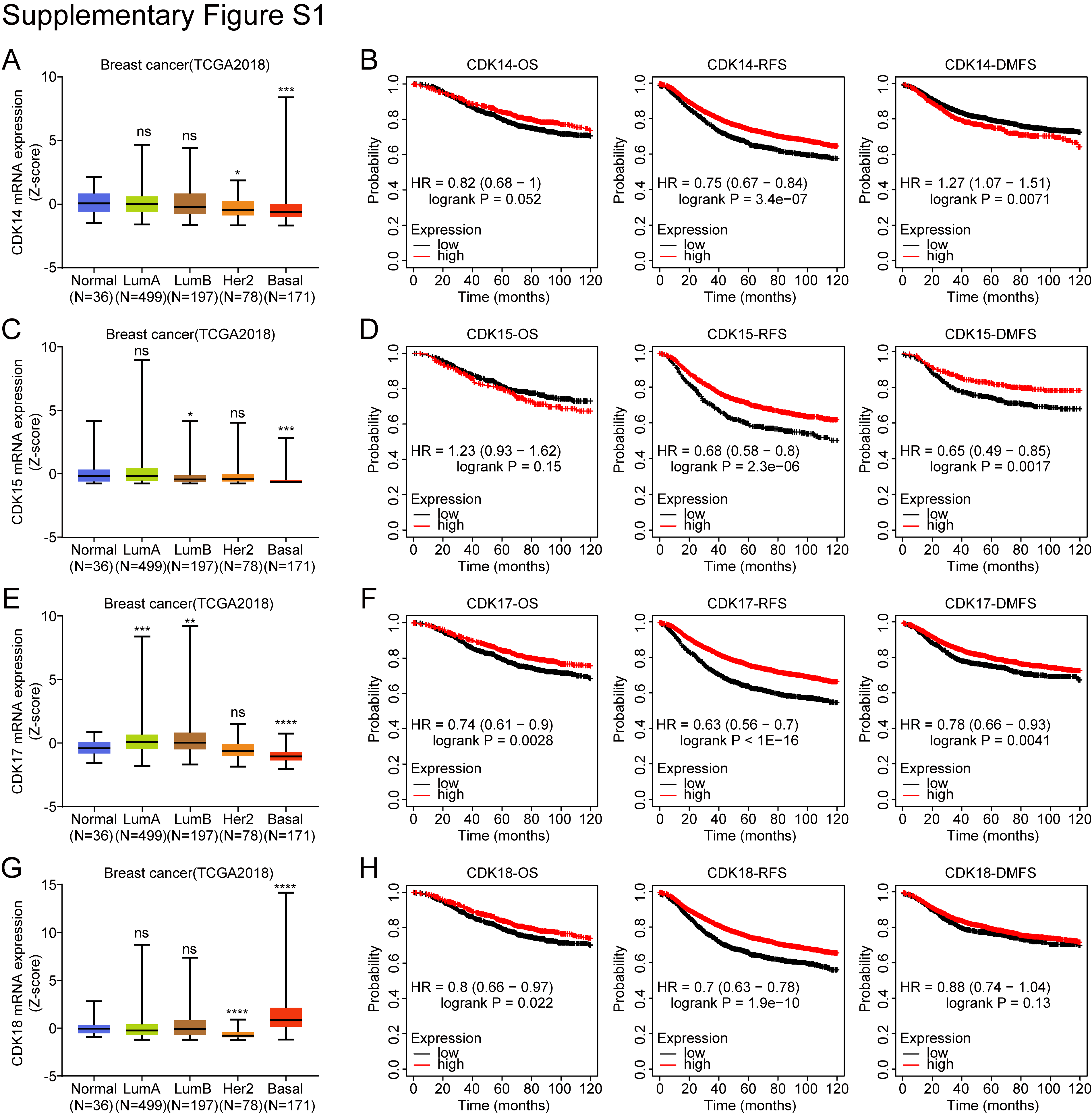

Supplement: Supplementary file 1 — Additional file 1: Supplementary Figure S1. Clinical relevance analysis of atypical CDKs (CDK14, 15, 17, 18) in breast cancer. A mRNA expression analysis of CDK14 in different subtypes of breast cancer using data from TCGA breast cancer dataset. B Survival analysis of overall survival (OS), recurrence-free survival (RFS), and distant metastasis-free survival (DMFS) for breast cancer patients with stratified mRNA expression of CDK14 by KM plotter. C mRNA expression analysis of CDK15 in different subtypes of breast cancer using data from TCGA breast cancer dataset. D Survival analysis of OS, RFS, and DMFS for breast cancer patients with stratified mRNA expression of CDK15 by KM plotter. E mRNA expression analysis of CDK17 in different subtypes of breast cancer using data from TCGA breast cancer dataset. F Survival analysis of OS, RFS, and DMFS for breast cancer patients with stratified mRNA expression of CDK17 by KM plotter. G mRNA expression analysis of CDK18 in different subtypes of breast cancer using data from TCGA breast cancer dataset. H Survival analysis of OS, RFS, and DMFS for breast cancer patients with stratified mRNA expression of CDK18 by KM plotter. Data are presented as mean ± SD (A, C, E, and G). p values were obtained by two-tailed Student’s t-test (A, C, E, and G) or log rank test (others). All *p < 0.05, ** p < 0.01, ***p < 0.001, **** p < 0.0001, ns, not significant. [file 13046_2022_2362_MOESM1_ESM.tif]

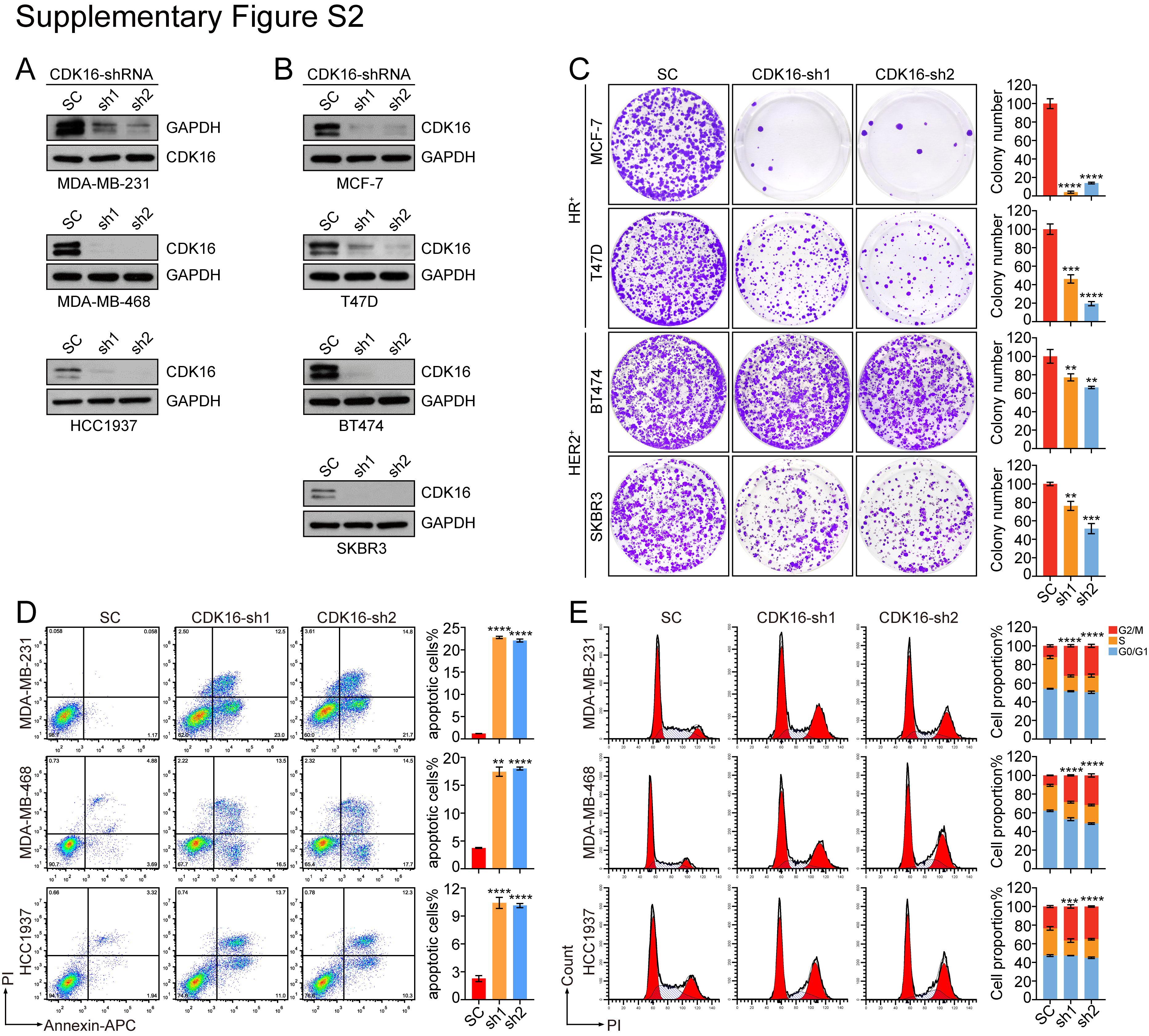

Supplement: Supplementary file 2 — Additional file 2: Supplementary Figure S2. CDK16 knockdown induces proliferation inhibition in TNBC and HR+ cells but not in HER2+ breast cancer cells. A-B Immunoblot analysis to verify CDK16 knockdown in indicated TNBC cells (A) and in HR+ and HER2+ breast cancer cells (B) transfected with two individual CDK16-shRNAs. C 2D-colony formation assay of indicated HR+ and HER2+ cells with CDK16-KD. Shown are representative images (scale bar, 100 μm, left panel) and quantification of colonies (right panel). D Apoptosis analysis of indicated TNBC cells with CDK16-KD. Shown are FACS results (left panel) and statistics of cells in early apoptosis (right panel). E Cell cycle analysis of indicated TNBC cells with CDK16-KD. Shown are FACS results (left panel) and statistics of cells in specific phases (right panel). Data are shown as mean ± SD and p values were obtained by two-tailed Student’s t-test. All *p < 0.05, ** p < 0.01, ***p < 0.001, **** p < 0.0001, ns, not significant. [file 13046_2022_2362_MOESM2_ESM.tif]

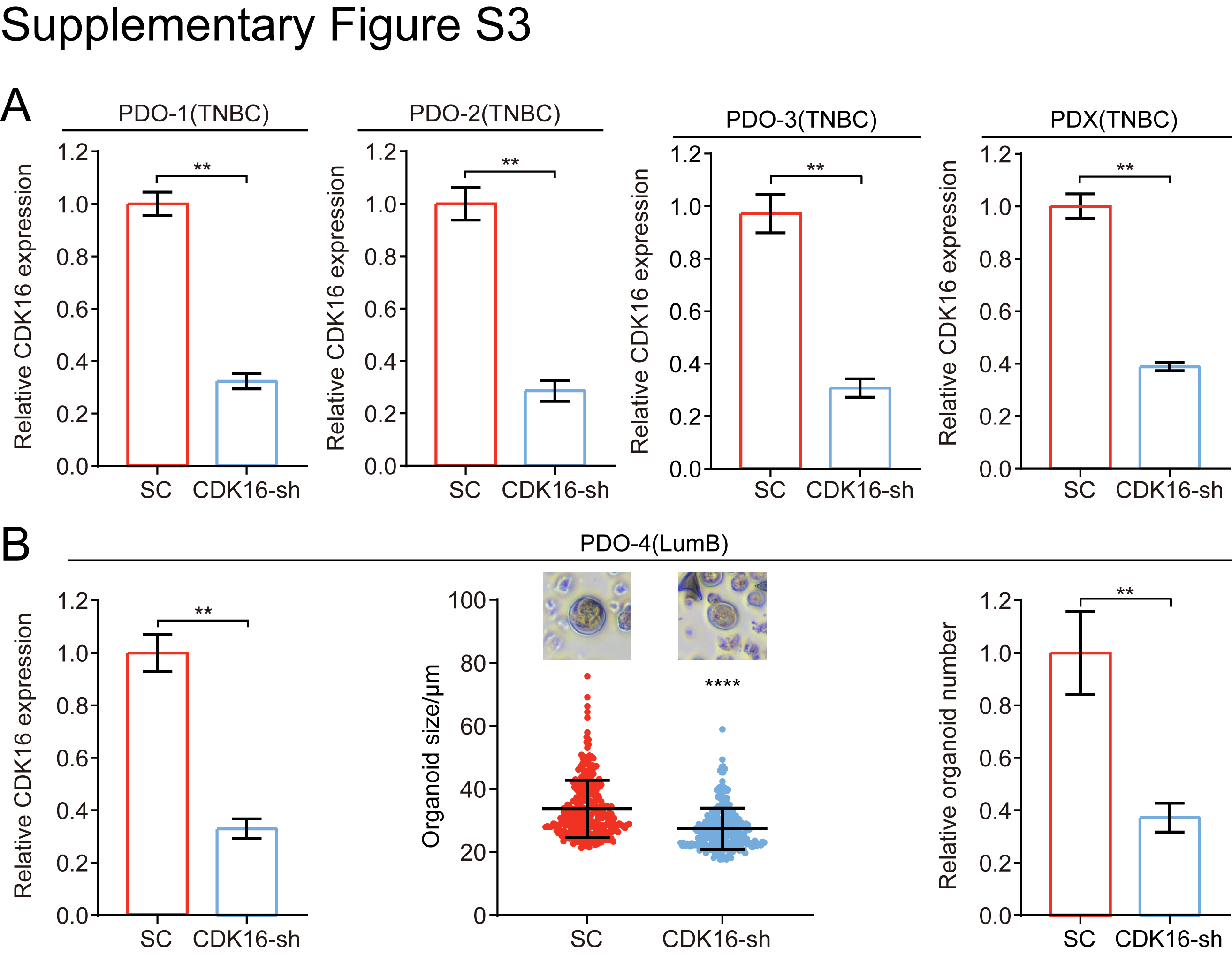

Supplement: Supplementary file 3 — Additional file 3: Supplementary Figure S3. Knockdown efficiency of CDK16 in PDO and PDX models. A qPCR analysis to verify CDK16 knockdown in three independent TNBC PDOs and the PDX model. B Organoid formation assay for the established LumB PDO model when CDK16 was knocked down. Shown are qPCR analysis to verify CDK16 knockdown (left), statistics of tumor organoid size (middle), and relative number of formed organoids (right). Data are presented as mean ± SD and p values were obtained by two-tailed Student’s t-test. All *p < 0.05, ** p < 0.01, ***p < 0.001, **** p < 0.0001, ns, not significant. [file 13046_2022_2362_MOESM3_ESM.tif]

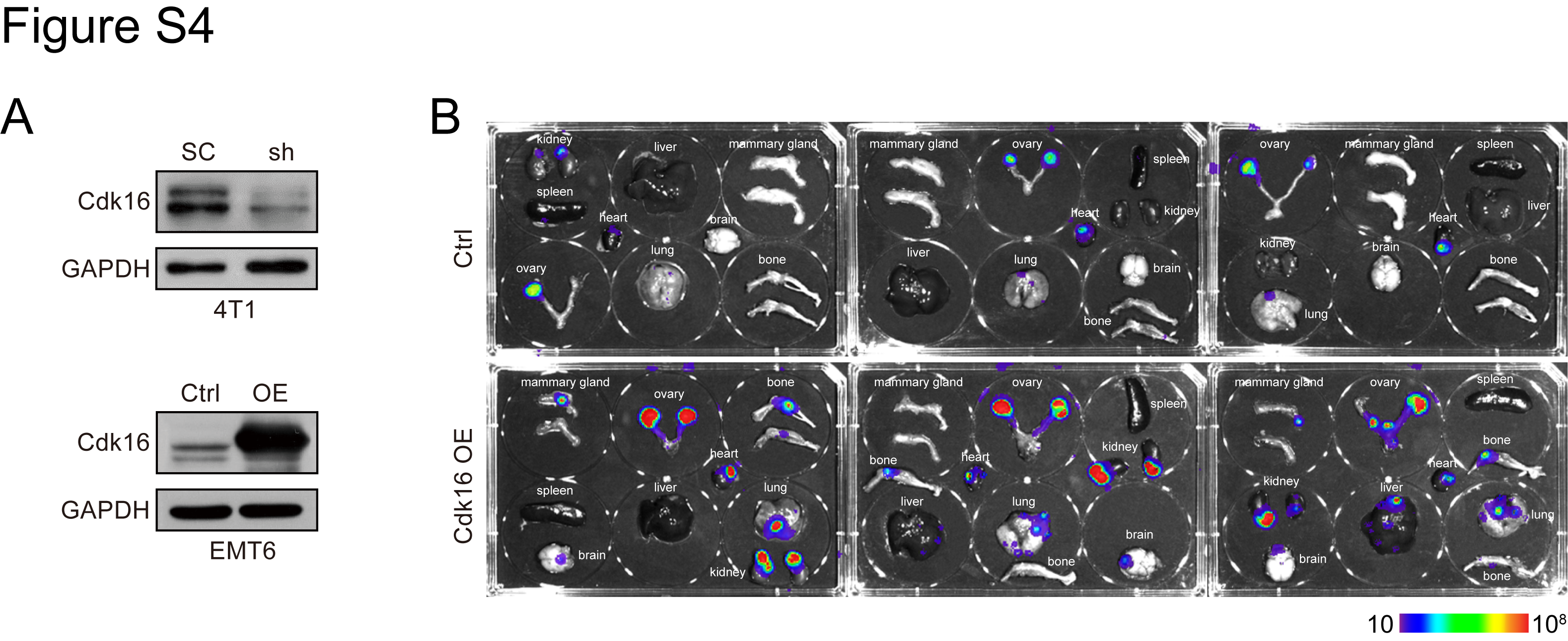

Supplement: Supplementary file 4 — Additional file 4: Supplementary Figure S4. Cdk16 promotes tumor metastasis of mouse TNBC in systemic metastasis models. A Immunoblot analysis to verify Cdk16 knockdown in 4T1 cells and Cdk16 overexpression in EMT6 cells. B BLI images of major organs dissected from mice bearing control or Cdk16-OE EMT6 cells showed the metastatic sites of tumor cells. [file 13046_2022_2362_MOESM4_ESM.tif]

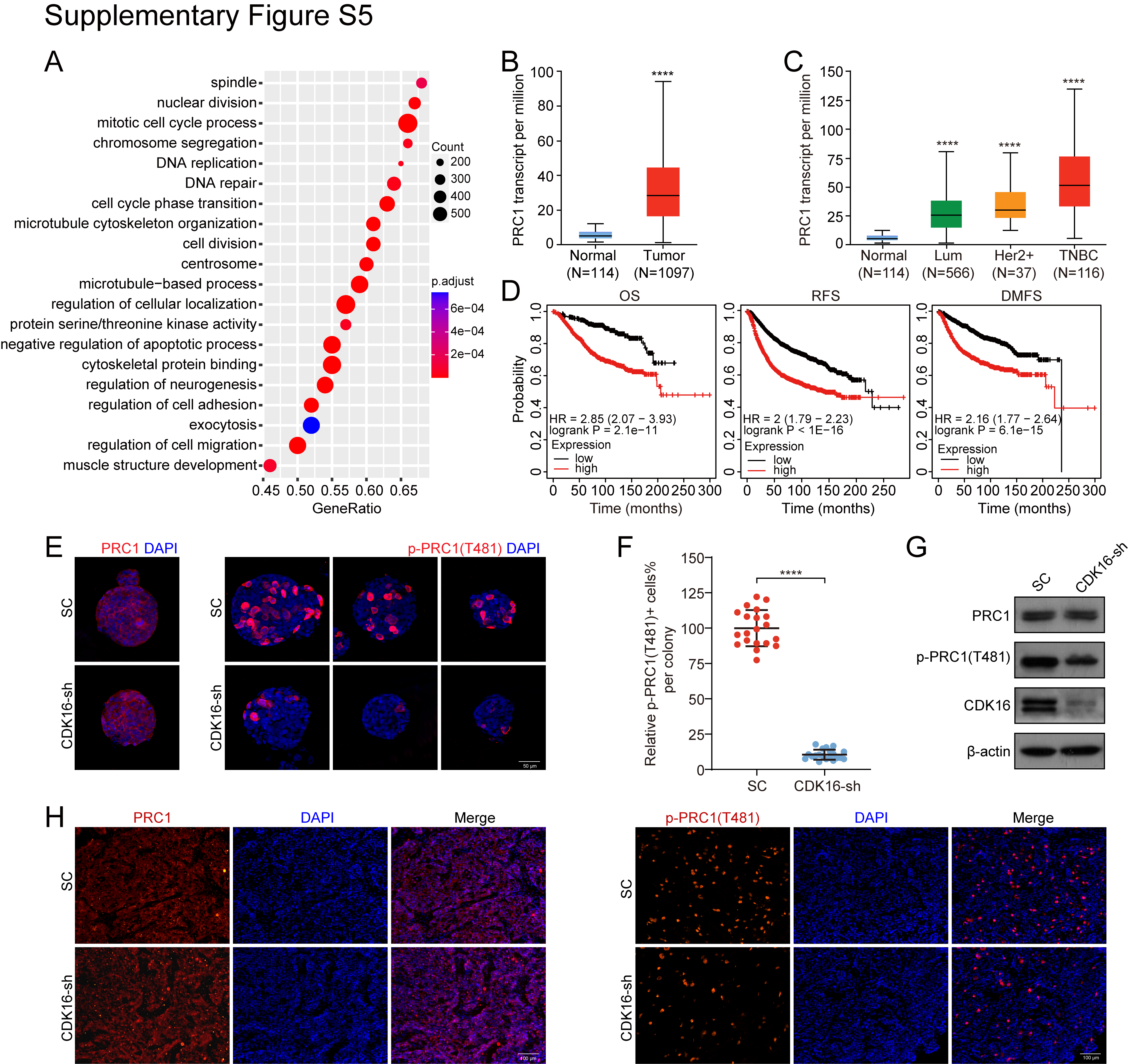

Supplement: Supplementary file 5 — Additional file 5: Supplementary Figure S5. CDK16 regulates tumor growth of TNBC by phosphorylating PRC1. A GO analysis of down-regulated genes in CDK16-KD MDA-MB-231 cells according to RNA-seq data. B-C PRC1 mRNA expression in normal and breast cancer tissues (B), and in different subtypes (C) of TCGA samples in the ULCAN database. D Survival analysis of OS, RFS, and DMFS for breast cancer patients with low or high mRNA expression of PRC1 by KM plotter. E-F IF analysis for PRC1 and p-PRC1(T481) of control and CDK16-KD organoids in established PDO model. Shown are representative IF images (E) and statistics of the proportion of p-PRC1(T481) positive cells per organoid (F). G Immunoblot analysis for PRC1 and p-PRC1(T481) expression in control and CDK16-KD organoids formed in (E). H IF analysis of control and CDK16-KD PDX tumor sections for PRC1 (left panel) and p-PRC1(T481) (right panel) followed by DAPI staining. Data are presented as mean ± SD (B, C, and F). p values were obtained by unpaired two-tailed t test (B, C, and F) or log rank test (D). All *p < 0.05, ** p < 0.01, ***p < 0.001, **** p < 0.0001, ns, not significant. [file 13046_2022_2362_MOESM5_ESM.tif]

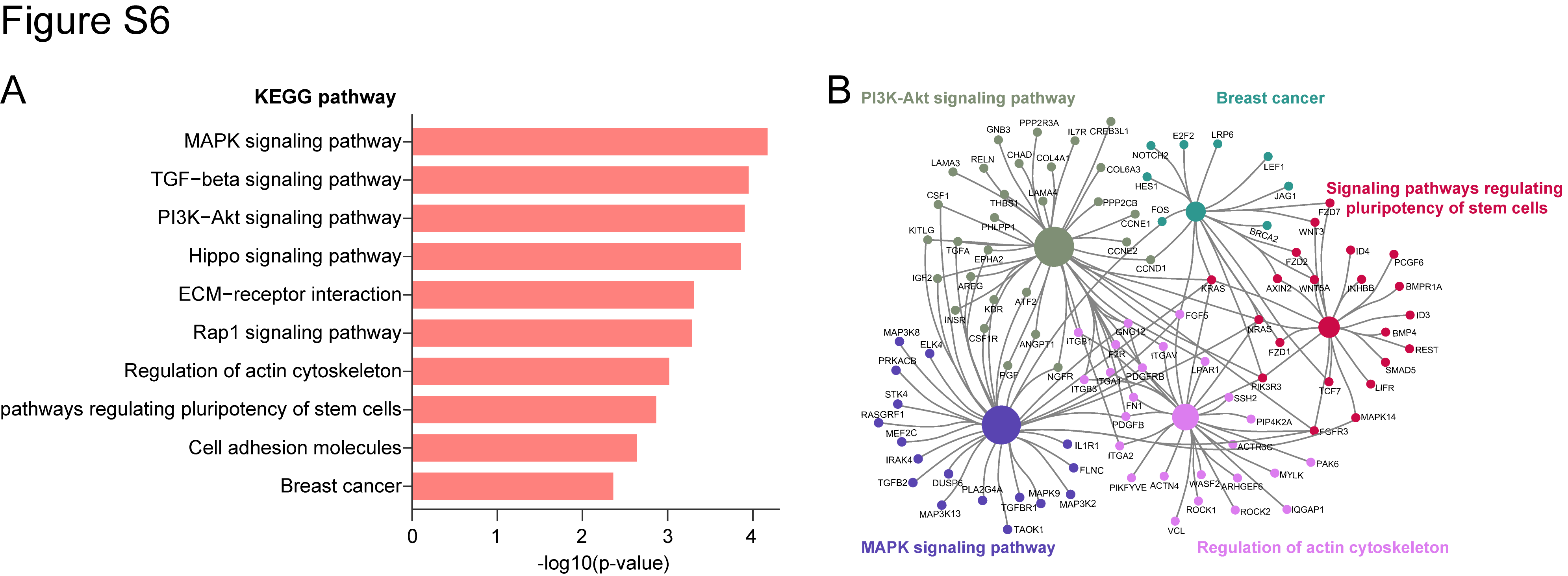

Supplement: Supplementary file 6 — Additional file 6: Supplementary Figure S6. CDK16 inhibition is involved in multiple cancer-related signaling pathways. A KEGG analysis of the significantly decreased pathways in CDK16-KD MDA-MB-231 cells. B Network and key genes of the representative pathways enriched in the KEGG analysis. [file 13046_2022_2362_MOESM6_ESM.tif]
