# Supplementary material for: CDK16 promotes the progression and metastasis of triple-negative breast cancer by phosphorylating PRC1
Source: J Exp Clin Cancer Res. 2022 Apr 21;41:149. doi: 10.1186/s13046-022-02362-w (PMC9027050; doi:10.1186/s13046-022-02362-w)
Supplement: Supplementary file 7 — Additional file 7: Supplementary Table S1. List of primers used in qPCR analysis. [file 13046_2022_2362_MOESM7_ESM.docx]

**Supplementary Table S1.** List of primers used in qPCR analysis.

| Gene | Forward (5’-3’) | Reverse (5’-3’) |
| --- | --- | --- |
| GAPDH | GGAGCGAGATCCCTCCAAAAT | GGCTGTTGTCATACTTCTCATGG |
| CDK16 | TCCGTCGTGTCAGCCTATCT | TCATGTTCCAGTCTGATCTCCTT |
| PRC1 | AAGTGGAATTGATGCGAAAACAG | CCTCACGCCTAGAAGCCTTTG |
| CCNB1 | AACTTTCGCCTGAGCCTATTTT | TTGGTCTGACTGCTTGCTCTT |
| CDC2 | AAACTACAGGTCAAGTGGTAGCC | TCCTGCATAAGCACATCCTGA |
| CDC25C | AAGTGGCCTATATCGCTCCC | CCCTGGTTAGAATCTTCCTCCA |
| p21 | CGATGGAACTTCGACTTTGTCA | GCACAAGGGTACAAGACAGTG |
| p27 | AACGTGCGAGTGTCTAACGG | CCCTCTAGGGGTTTGTGATTGT |
| Bax | CCCGAGAGGTCTTTTTCCGAG | CCAGCCCATGATGGTTCTGAT |
| CDK2 | CCAGGAGTTACTTCTATGCCTGA | TTCATCCAGGGGAGGTACAAC |
| RRM2 | GTGGAGCGATTTAGCCAAGAA | CACAAGGCATCGTTTCAATGG |
| TOP2A | TTAATGCTGCGGACAACAAACA | CGACCACCTGTCACTTTCTTTT |
| MKI67 | ACGCCTGGTTACTATCAAAAGG | CAGACCCATTTACTTGTGTTGGA |
| MCM7 | GCCTGTGGGAAATATCCCTCG | GTACCACCTGTCGGAACCC |
