# Supplementary material for: CDK16 promotes the progression and metastasis of triple-negative breast cancer by phosphorylating PRC1
Source: J Exp Clin Cancer Res. 2022 Apr 21;41:149. doi: 10.1186/s13046-022-02362-w (PMC9027050; doi:10.1186/s13046-022-02362-w)
Supplement: Supplementary file 8 — Additional file 8: Supplementary Table S2. List of the antibodies used and their application. [file 13046_2022_2362_MOESM8_ESM.docx]

**Supplementary Table S2.** List of the antibodies used and their application.

| Antibody |  | Source | Catalog | Brand |
| --- | --- | --- | --- | --- |
| CDK16 | WB | Rabbit polyclonal | #4852 | Cell Signaling Technology, USA |
| Rb | WB | Mouse monoclonal | #9309 | Cell Signaling Technology, USA |
| p-Rb (S780) | WB | Rabbit monoclonal | #8180 | Cell Signaling Technology, USA |
| p-Rb (S807/811) | WB | Rabbit monoclonal | #8516 | Cell Signaling Technology, USA |
| PRC1 | WB | Rabbit monoclonal | ab51248 | Abcam, UK |
| p-PRC1 (T481) | WB | Rabbit monoclonal | ab62366 | Abcam, UK |
| GAPDH | WB | Mouse monoclonal | sc-32233 | Santa Cruz Biotechnology, USA |
| β-actin | WB | Mouse monoclonal | 66009-1 | Proteintech Group, USA |
| Lamin B1 | WB | Rabbit monoclonal | ab229025 | Abcam, UK |
| HRP IgG | WB | Goat anti-mouse | GTX213111-01 | GeneTex, USA |
| HRP IgG | WB | Goat anti-rabbit | GTX213110-01 | GeneTex, USA |
| PRC1 | IF | Rabbit monoclonal | ab51248 | Abcam, UK |
| p-PRC1 (T481) | IF | Rabbit monoclonal | ab62366 | Abcam, UK |
| α-Tubulin | IF | Mouse monoclonal | #3873 | Cell Signaling Technology, USA |
| Pericentrin | IF | Rabbit monoclonal | ab220784 | Abcam, UK |
| Ki67 | IF | Mouse monoclonal | #9449 | Cell Signaling Technology, USA |
| Cleaved caspase-3 | IF | Rabbit monoclonal | #9664 | Cell Signaling Technology, USA |
| Cy3 IgG | IF | Donkey anti-mouse | 715-165-150 | Jackson ImmunoResearch, USA |
| Cy3 IgG | IF | Donkey anti-rabbit | 711-165-152 | Jackson ImmunoResearch, USA |
| Cy5 IgG | IF | Donkey anti-rabbit | 711-175-152 | Jackson ImmunoResearch, USA |
| Alexa Flour 488 IgG | IF | Donkey anti-rabbit | A21206 | Thermo Fisher Scientific, USA |
| Alexa Flour 488 IgG | IF | Donkey anti-mouse | A21202 | Thermo Fisher Scientific, USA |
